# Endometrial small extracellular vesicles regulate human trophectodermal cell invasion by reprogramming the phosphoproteome landscape

**DOI:** 10.3389/fcell.2022.1078096

**Published:** 2022-12-22

**Authors:** Monique Fatmous, Alin Rai, Qi Hui Poh, Lois A. Salamonsen, David W. Greening

**Affiliations:** ^1^ Baker Heart and Diabetes Institute, Melbourne, VIC, Australia; ^2^ Department of Microbiology, Anatomy, Physiology and Pharmacology, La Trobe University (LTU), Melbourne, VIC, Australia; ^3^ Central Clinical School, Monash University, Melbourne, VIC, Australia; ^4^ Baker Department of Cardiometabolic Health, University of Melbourne, Melbourne, VIC, Australia; ^5^ Baker Department of Cardiovascular Research, Translation and Implementation, LTU, Melbourne, VIC, Australia; ^6^ Department of Biochemistry and Chemistry, LTU, Melbourne, VIC, Australia; ^7^ Centre for Reproductive Health, Hudson Institute of Medical Research, Clayton, VIC, Australia; ^8^ Department of Molecular and Translational Medicine, Monash University, Clayton, VIC, Australia

**Keywords:** trophoblast invasion, extracellular vesicles, proteomics, phosphoproteomics, surfaceome, trophectoderm cells

## Abstract

A series of cyclical events within the uterus are crucial for pregnancy establishment. These include endometrial regeneration following menses, under the influence of estrogen (proliferative phase), then endometrial differentiation driven by estrogen/progesterone (secretory phase), to provide a microenvironment enabling attachment of embryo (as a hatched blastocyst) to the endometrial epithelium. This is followed by invasion of trophectodermal cells (the outer layer of the blastocyst) into the endometrium tissue to facilitate intrauterine development. Small extracellular vesicles (sEVs) released by endometrial epithelial cells during the secretory phase have been shown to facilitate trophoblast invasion; however, the molecular mechanisms that underline this process remain poorly understood. Here, we show that density gradient purified sEVs (1.06–1.11 g/ml, Alix^+^ and TSG101^+^, ∼180 nm) from human endometrial epithelial cells (hormonally primed with estrogen and progesterone vs. estrogen alone) are readily internalized by a human trophectodermal stem cell line and promote their invasion into Matrigel matrix. Mass spectrometry-based proteome analysis revealed that sEVs reprogrammed trophectoderm cell proteome and their cell surface proteome (surfaceome) to support this invasive phenotype through upregulation of pro-invasive regulators associated with focal adhesions (NRP1, PTPRK, ROCK2, TEK), embryo implantation (FBLN1, NIBAN2, BSG), and kinase receptors (EPHB4/B2, ERBB2, STRAP). Kinase substrate prediction highlighted a central role of MAPK3 as an upstream kinase regulating target cell proteome reprogramming. Phosphoproteome analysis pinpointed upregulation of MAPK3 T204/T202 phosphosites in hTSCs following sEV delivery, and that their pharmacological inhibition significantly abrogated invasion. This study provides novel molecular insights into endometrial sEVs orchestrating trophoblast invasion, highlighting the microenvironmental regulation of hTSCs during embryo implantation.

## Introduction

Embryo implantation is dictated by a series of endometrial events and regulated signaling from the endometrium to the incoming embryo (pre-implantation blastocyst) (Evans et al., 2016; Aplin and Ruane, 2017). Following menstruation, the functional layer of the endometrium is re-constructed from stem cells in the underlying basal layer under the influence of maternal hormone estrogen (as estradiol 17β, E) during which the endometrium is in a non-receptive state. Following ovulation, progesterone (P) released by ovaries (corpus luteum), in conjunction with E, reprograms the endometrium towards a secretory phase marked by released of pro-implantation factors {cytokines [e.g., leukemia inhibitory factor (LIF), IL-11] and growth factors (e.g., EGF)} including extracellular vesicles that act on blastocyst to promote implantation (Salamonsen et al., 2016; Salamonsen et al., 2021). Implantation begins with the apposition (correct positioning) and attachment of outer trophectodermal cells of the blastocyst to the maternal endometrial epithelium (Salamonsen et al., 2021). This is followed by invasion of the trophectodermal cells through the endometrial luminal epithelium, after which they differentiate into trophoblast cell lineages, cyto-, syncytial- and extravillous (Aplin and Ruane, 2017). The latter traffic through the endometrial decidua and some invade the spiral arterioles which they transform into flaccid sacs ideal for blood and nutrient exchange. Thus, *in vivo* acquisition of the invasive phenotype is critical for placental formation to enable adequate supply of oxygen and nutrients to the developing embryo, and regulating maternofetal immune tolerance (Adamson et al., 2002; Prefumo et al., 2006; Huang et al., 2008; Abumaree et al., 2012; Wang et al., 2012; Delorme-Axford et al., 2013; McConkey et al., 2016; Pollheimer et al., 2018; Ander et al., 2019; Sung et al., 2022). Failure of any of these processes to proceed normally can result in several complications and diseases of pregnancy such as recurrent miscarriage, preeclampsia, and intrauterine growth restriction (Dey et al., 2004; Lyall et al., 2013; Jarvis, 2016; Kim and Kim, 2017; Melchiorre et al., 2022).

To achieve endometrial invasion, trophectodermal cells must regulate regional endometrial cell junctions and invade through the underlying basement membrane (Aplin and Ruane, 2017). Our understanding of the molecular mechanisms regulating these processes is still emerging with accumulating evidence highlighting the central role paracrine factors secreted by endometrium play (Evans et al., 2016; Huppertz, 2019). Within this milieu of paracrine factors, membranous particles released by cells called extracellular vesicles (EVs), mainly a major sub-class of EVs called small EVs (sEVs, 50–200 nm in size) are also emerging as important mediators (Greening et al., 2016; Simon et al., 2018; Gurung et al., 2020) of trophoblast invasion (Liu et al., 2020a; Ding et al., 2021). sEVs transfer functional cargo (including proteins and nucleic acids) to target cells to regulate the cellular adhesion network and signaling pathways, and to reprogram trophectodermal and trophoblast cells to support embryo implantation (Evans et al., 2019). sEVs from mesenchymal stem cells functionally transfer microRNA let-7b to promote trophoblast invasion *via* FOXO1 (Chen et al., 2020) and activation of ERK/MMP-2 pathway (Liu et al., 2020b). Further, sEVs containing miR-486-5p from human placental microvascular endothelial cells regulate proliferation and invasion of trophoblasts *via* targeting IGF1 (Ma et al., 2021). Dysregulation of EV-mediated signalling from placental tissue and trophoblast cells is further associated with complications such as preeclampsia (Cheng et al., 2019; Wang et al., 2020).

Endometrial epithelial cells are the prime source of EVs in uterine fluid that signal to the pre-implantation embryo (Rai et al., 2021a). We have previously shown that human endometrial EVs isolated from human uterine fluid from the secretory phase (EP) vs. proliferative phase (E) promote human trophectodermal stem cell (hTSC) invasion through extracellular matrix (Rai et al., 2021a). However, the underlying mechanism and signalling pathways remain unknown. In the current study, using mass spectrometry-based proteomics and phosphoproteomics, we demonstrate that EP-regulated endometrial cell-derived sEVs (but not E-regulated sEVs) promote hTSCs invasion *via* MAPK activation and that pharmacological inhibition of MAPK activation abrogates this process.

## Results

### Hormonal regulation of sEV proteome released by endometrial cells underscore human trophectoderm cell invasion

**FIGURE 1:**
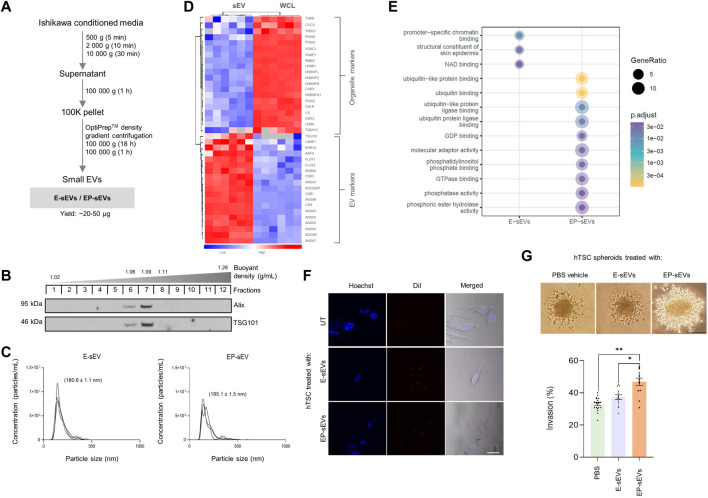
Small extracellular vesicles from endometrial cells representing secretory phase promote human trophectoderm cell invasion**. (A)** Experimental workflow for isolation of endometrial sEVs from Ishikawa cells using differential ultracentrifugation and buoyant density-based separation (OptiPrep™). Ishikawa cells were primed with estrogen (E) (proliferative phase), and subsequent E and progesterone (P) (EP; secretory phase) to derive E-sEVs and EP-sEVs. **(B)** Western blot analysis of density-gradient purified sEVs (fractions 6–8, 1.06–1.11 g/ml) using anti- Alix and -TSG101; EP-sEVs. **(C)** Size distribution and concentration (particles/mL) of E- and EP-sEVs by single nanoparticle analysis (*n* = 3). **(D)**. Quantitative mass spectrometry proteome analysis of Ishikawa cell (WCL; combined E/EP-priming) and sEVs (combined E/EP priming), *n* = 6. Hierarchical clustering of protein expression of EV (sEV/exosome) marker proteins and cell organelle markers (i.e., nucleus, mitochondria, endoplasmic reticulum and cytoskeleton). Scale represents normalized label-free quantitation intensity (*p* < 0.05, Student’s T. test). **(E)** Gene Ontology enrichment analysis (DAVID) of EP-sEVs vs. E-sEVs (significantly enriched or uniquely identified). **(F)** Uptake of E-/EP-sEVs to hTSCs using live fluorescence and bright-field microscopic analysis (100 μg/ml, 2 h). sEVs stained with lipophilic tracer DiI (red), nuclei stained with Hoechst (blue). Scale bar, 20 µm. **(G)** hTSC spheroid Matrigel™ invasion assay; top panel, phase-contrast microscopy images of hTSC spheroids pre-treated with E-/EP-sEVs or PBS (untreated; UT) and overlaid on Matrigel™ matrix to invade (48 h), scale bar, 100 μm; bottom panel, mean invasive outgrowth ±SEM (*n* = 13–20 replicates with four invasive fronts measured per spheroid, *p ≤ .001, **p ≤ .0001).

To recapitulate hormonal priming of the endometrium, we primed human Ishikawa endometrial epithelial cells with E to mimic the proliferative phase, evident from progesterone receptor (PR) A/B upregulation (Supplementary Figure S1). This was followed by sequential stimulation with EP to mimic the secretory phase, evident by down-regulated expression of the PR receptor in a negative feedback loop mechanism as occurs *in vivo* (Lessey et al., 1988; Lessey et al., 1996) (Supplementary Figure S1). Next, endometrial E-/EP-sEVs were isolated and purified using differential ultracentrifugation coupled to density gradient-based separation (Rai et al., 2021b) (Figure 1A).

In accordance with International Society for Extracellular Vesicles (ISEV) research guidelines (Thery et al., 2018), we characterized sEVs for their biophysical and biochemical properties; sEVs displayed 1.06–1.11 g/ml buoyant density, were positive for stereotypic sEV markers ALIX and TSG101 (Figure 1B) and were ∼140–150 nm diameter based on single particle tracking analysis [below size range for small EVs, as defined by MISEV (Thery et al., 2018)] (Figure 1C) (E-sEVs: mean 142.8 ± 1.1 nm, average particle concentration/ml: 8.21E+08; EP-sEVs: mean 151.3 ± 9.5 nm, average particle concentration/ml: 6.62E + 08). We next performed proteome profiling of sEVs and their parental cells using nLC-MS/MS and data-dependent acquisition (Rai et al., 2021c; Kompa et al., 2021). We found that the proteomes of parental cells *versus* their derived sEVs (fraction 6–8, 1.06–1.11 g/ml) were distinct (Supplementary Figure S2), with sEVs compared to cells significantly enriched in sEV markers (e.g., CD9/63/81, Alix/PDCD6IP, TSG101) (Greening et al., 2017; Rai et al., 2021d; Mathieu et al., 2021), which includes CD63 as well as proposed universal marker of sEVs/exosomes, SDCBP (Kugeratski et al., 2021) (Figure 1D, Supplementary Table S1) (Rai et al., 2021c; Poh et al., 2021). Further, we show sEVs display lower abundance for non-EV (intracellular/organelle) proteins (e.g., nucleus; HNRNPC, mitochondria; CYSC, endoplasmic reticulum; CALR) (Poh et al., 2021) (Figure 1D, Supplementary Table S1). Using these multiple sEV inclusion proteins to verify the presence of sEVs, is in direct agreement with MISEV guidelines (Thery et al., 2018). These data collectively support successful enrichment of purified sEVs.

We further compared the proteomes of sEVs isolated at different cycle phases (E and EP) to gain insight into their potential role in embryo implantation, particularly trophectoderm/trophoblast invasion. There were striking differences in E- and EP-sEV proteomes (Supplementary Table S2; Supplementary Figure S3) with significantly abundant proteins in EP-sEVs (243 proteins) implicated in cell-cell adhesion, migration, invasion and embryo development (Greening et al., 2016), human embryo implantation (Enciso et al., 2018; Matorras et al., 2018) and endometrial receptivity (Diaz-Gimeno et al., 2011; Altmae et al., 2017; Azkargorta et al., 2018) (Supplementary Figure S3). Additionally, several proteins (DDAH2, PGAM1) were identified in human uterine fluid collected from the secretory phase (Rai et al., 2021a) (Supplementary Figure S3). Further, comparative analysis of Gene Ontology functional enrichment of EP-sEVs (*versus* E-sEVs) revealed distinct molecular and enzymatic functions associated with ubiquitin binding/interaction, phosphatase activity/binding, molecular adaptor activity, GTPase activity, and ligase binding (Figure 1E, Supplementary Table S3).

Comparative proteome analysis of EP-sEVs (*versus* E-sEVs) revealed molecular players of epithelial cell migration (BMPR2, DDR1, IGSF8, MST1R), embryo development (COPS3, CUL3, NOTCH1, PLCG1, ADAM10), cell-cell adhesion (SDCBP, EPCAM, NOTCH1), nitric oxide biosynthesis (e.g., DDAH2, AKT1 and SPR) and cell invasion (CSTB, DDR1, RAB25, ST14, TXN) (Supplementary Figure S3; Supplementary Table S2). Importantly, EP-sEVs are enriched in key players shown to remodel endometrium to promote receptivity, or embryo to promote implantation processes (Illera et al., 2000; Haslinger et al., 2013; Zhao et al., 2018; Segura-Benitez et al., 2022); enriched in regulators of endometrial receptivity (CTNNB1, EGFR, EPCAM, NOTCH1 and DDRI), as well as promote implantation processes (BMPR2, BTF3, COPS3, PLCG1, RAB14, ADAM10, CUL3, ITGAV and AKT1) (Table 1). This further supports the successful hormonal priming of endometrial epithelial cells, the distinct molecular composition of sEVs, and highlights the pro-invasive function of EP-primed endometrial cell sEVs. Moreover, similar proteome reprogramming was also observed in sEVs from the human ECC1 endometrial epithelial cells following the same hormonal treatment (Greening et al., 2016) (Supplementary Figure S4).

**TABLE 1:** Hormonal regulation of endometrial sEV proteome reprograms composition to support trophectoderm cell function and implantation.

^a^Uniprot accession	^a^Gene name	^a^Protein name	^b^Diff. Expression log_2_ (EP/E)	Implicated function	References
O14672	ADAM10	Disintegrin and metalloproteinase domain-containing protein 10	0.79	Proteolytic activity which promotes cell migration. May regulate adhesion response	Kim et al. (2006)
P31749	AKT1	RAC-alpha serine/threonine-protein kinase	0.49	Enables trophoblast migration in response to epidermal growth factor signalling	Haslinger et al. (2013)
Q13873	BMPR2	Bone morphogenetic protein receptor type-2	EP unique	Involved in BMP signalling for the development of extraembryonic cell lineages for the pre-implantation embryo. BMP2 signalling recently shown to promote human trophoblast invasion	Graham et al., 2014 Zhao et al., 2018
P20290	BTF3	Transcription factor BTF3	EP unique	Potentially regulates ERα transcription. Required for post-implantation embryonic development	Ding et al., 2019 Deng and Behringer, (1995)
Q9UNS2	COPS3	COP9 signalosome complex subunit 3	EP unique	Integral part of COP9 signalosome complex, required to maintain embryonic epiblast cell survival and development of early embryo	Yan et al. (2003)
P35222	CTNNB1	Catenin beta-1	0.39	Required for proper uterine development. Depletion results in impaired decidualisation and endometrial receptivity	Jeong et al., 2009 Zhou et al., 2020
Q13618	CUL3	Cullin-3	EP unique	Modulation of CUL3/β-catenin pathway promotes endometrial receptivity and supports attachment of trophectoderm cells to endometrial epithelium	Huang et al. (2020)
Q08345	DDR1	Epithelial discoidin domain-containing receptor 1	EP unique	Regulates endometrial cell proliferation by inactivating endothelin-1, to stimulate AKT phosphorylation and DNA synthesis in stromal cells	Houshdaran et al. (2014)
P00533	EGFR	Epidermal growth factor receptor	0.81	Regulates endometrial decidualisation through BMP2 and WNT4 downstream effectors. Ablation of EGFR prevents stromal epithelial to mesenchymal transition	Large et al. (2014)
P16422	EPCAM	Epithelial cell adhesion molecule	1.63	Cell surface adhesion molecule that maintains epithelial integrity through modulation of E-cadherin. Expression of EPCAM temporally regulated to balance maintenance of epithelial integrity with endometrial receptivity	Poon et al. (2015)
P06756	ITGAV	Integrin alpha-V	0.21	Dimerises with integrin β3 to form an αvβ3 receptor that promotes endometrial receptivity and potentially coordinates embryo adhesion for implantation	Illera et al., 2000, Segura-Benitez et al., 2022
P46531	NOTCH1	Neurogenic locus notch homolog protein 1	EP unique	Maintains endometrial integrity during window of implantation	Afshar et al. (2012)
P19174	PLCG1	1-phosphatidylinositol 4,5-bisphosphate phosphodiesterase gamma-1	EP unique	Signal transduction molecule in response to tyrosine kinase signalling, that is required for proliferation of embryonic cell types for normal development	Ji et al. (1997)
P61106	RAB14	Ras-related protein Rab-14	1.2	KIF16B/Rab14 complex regulates surface expression of FGFR2 and fibroblast growth factor signal transduction (essential for embryogenesis) through golgi-to-endosome trafficking of vesicles expression FGFR	Ueno et al. (2011)

^a^ Uniprot Accession number, gene name and protein description annotated from UniProt: https://www.uniprot.org/

^b^ Differential expression using Log2 (EP-sEV/E-sEV). Fold change (FC) not calculated for unique protein IDs.

We next questioned whether these sEVs could be taken up by human embryo-derived TSCs; for this we employed a previously reported human trophoblast stem cell line (hTSC) established from individual blastomeres of donated human embryos (Zdravkovic et al., 2015; Evans et al., 2019; Poh et al., 2021). Confocal microscopy revealed that sEVs labelled with lipophilic dye DiI (Rai et al., 2021a) were readily taken up by hTSCs within 2 h (Figure 1F, Supplementary Figure S5). To assess their ability to regulate trophectoderm cell invasion, we generated spheroids of hTSCs [∼1200 cells as blastocyst mimetics (Evans et al., 2019; Evans et al., 2020a)], stimulated these with sEVs and assessed their capacity to invade into Matrigel™ matrix. Consistent with previous reports for human ECC1-derived sEVs (Evans et al., 2019; Rai et al., 2021a), we demonstrate EP-sEV treated hTSC spheroids display significantly greater invasive outgrowth in Matrigel™ matrix compared to vehicle control (PBS treated) and E-sEV treated hTSC spheroids (Figure 1G, Supplementary Figure S6).

### Endometrial small extracellular vesicles reprogram trophectoderm cell proteome towards a pro-invasive phenotype

**FIGURE 2:**
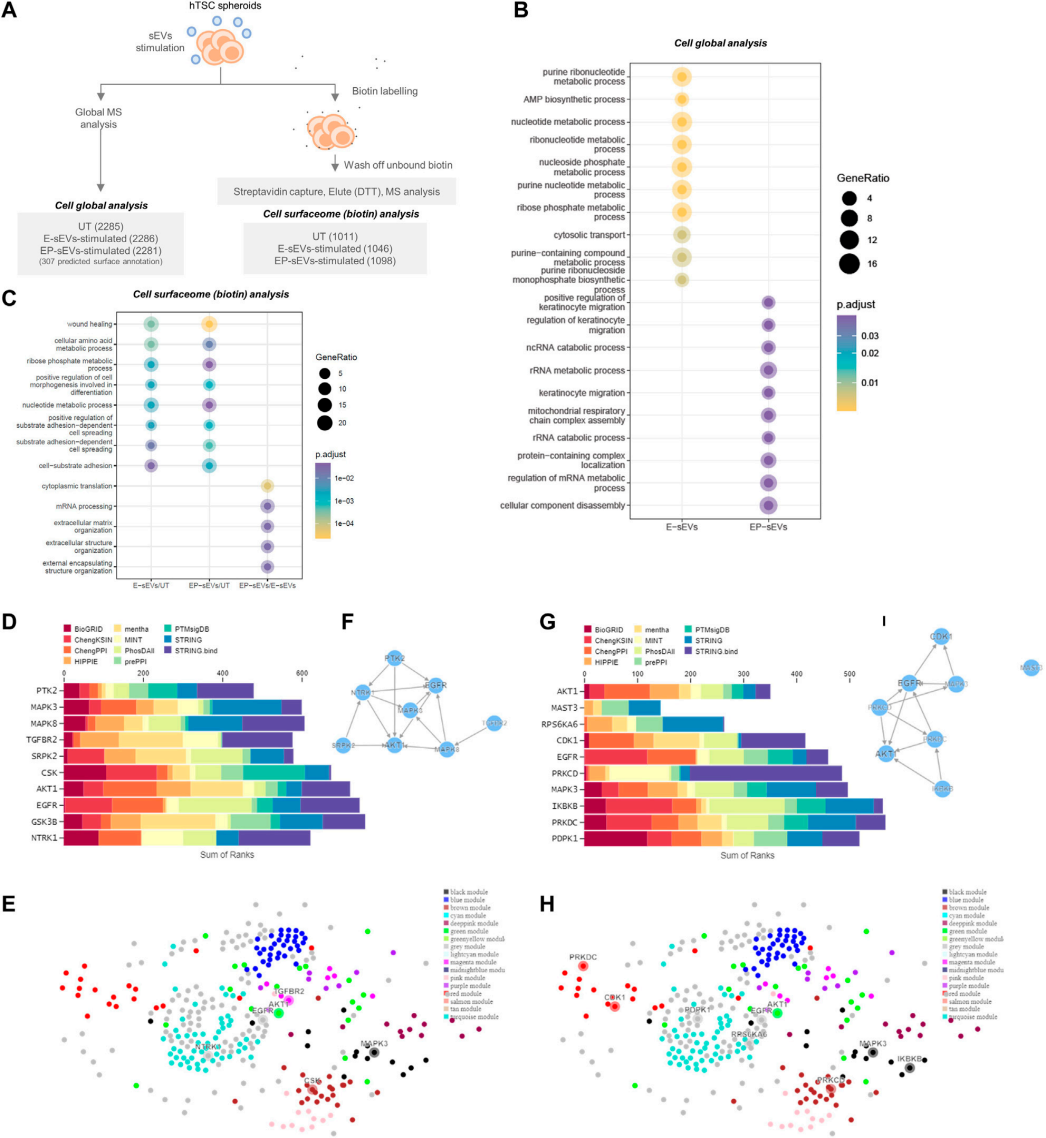
Endometrial small extracellular vesicles reprogram trophoblast proteome towards invasive phenotype. **(A)** Experimental workflow of hTSC proteome remodeling following treatment with E-/EP-sEV at global and cell surface proteome, with quantified proteins for each group shown. **(B)** Gene Ontology enrichment analysis (DAVID) for biological processes enriched in TSCs treated with either EP-sEVs or E-sEVs. **(C)** Gene Ontology enrichment analysis (DAVID) for biological processes enriched in proteins abundant in TSC cell surfaceome following treatment with E-/EP-sEVs (and UT). **(D–H)** Kinase Enrichment Analysis 3 (KEA3) (Kuleshov et al., 2021) was employed to identify upstream kinases whose substrates are overrepresented in differentially abundant proteins. For higher abundant proteins following EP-sEVs treatment **(D)** bar chart displays the top-ranking kinases across different libraries whose putative substrates are overrepresented **(E)** Human kinome regulatory network highlighting the top-ranked kinases with all kinases labelled by WGCNA modules and **(F)** Kinase co-regulatory networks constructed from top-ranked kinase enrichment results for various kinase-substrate interaction libraries. For higher abundant surface proteins in following EP-sEVs treatment, **(G)** bar chart displays the top-ranking kinases across different libraries whose putative substrates are overrepresented, **(H)** Human kinome regulatory network highlighting the top-ranked kinases with all kinases labelled by WGCNA modules and **(I)** Kinase co-regulatory networks constructed from top-ranked kinase enrichment results for various kinase-substrate interaction libraries.

To provide insights into the molecular changes in trophectoderm/trophoblast cells that support their sEV-driven invasion, we performed proteomic profiling of hTSCs following single-dose sEV treatment (Figure 2A). A total of 2,318 proteins were identified (Supplementary Table S4) of which 108 were either uniquely identified (55) or displayed significantly higher abundance (53, *p* < .05) in hTSCs treated with EP-sEVs compared to E-sEVs (Supplementary Table S5A/5B). Gene Ontology enrichment analysis (Biological Processes) of proteins distinct between EP-sEVs and E-sEVs identified significantly (*p* < .05) enriched terms such as “TGF-beta receptor signaling pathway; *p* < 4.62E-04”, “transmembrane receptor protein serine/threonine kinase signaling pathway; *p* < 5.11E-04,” “BMP signaling pathway; *p* < 6.65E-04,” “regulation of cell-substrate adhesion; *p* < 7.89E-04,” “keratinocyte cell migration; *p* < 3.59E-05”; processes that are implicated in cell invasion (Figure 2B, Supplementary Table S6). These proteins include migratory factors DPP4, ADAM9, and RALB, cell adhesion/invasive regulators CCN1, COL1A1, and THBS1, and cell motility signaling regulator NRP1 (Supplementary Table S7). Kinase Enrichment Analysis 3 (KEA3) (Kuleshov et al., 2021) was employed to identify upstream kinases whose substrates in hTSCs following EP-sEVs treatment were enriched, including EGFR, AKT1, and MAPK3 (Figure 2D). Human kinome regulatory network was used to highlight the top-ranked kinases with all kinases labelled by WGCNA modules, including various interaction networks associated with MAPK3 (Figures 2E, F).

Of note, 307 (from 2,318) proteins, based on cell surface databases CSPA/SURFY as cell surface proteins (plasma membrane, surface) (e.g., LAMP2, ADAM9, LTBP1, STT3A, DPP4, ITGA2/AV, ERO1A) (Supplementary Table S8) are known to interact with the ECM during invasion. Due to their essential role in ECM remodeling during invasion, we next ascertained their surface localization in hTSCs. For this, we employed cell membrane-impermeant biotin to capture surface proteins that were then identified and quantified using MS, similar to our previous report (Rai et al., 2021c) (Figure 2A, Supplementary Table S9).

A total of 1,346 surface proteins were found in hTSCs (vehicle-treated (UT), E-sEV-treated, EP-sEV-treated; identified in at least 2/3 biological replicates for each group, including 328 identified in CSPA/SURFY (Supplementary Table S10). Of the proteins identified as cell surface from CSPA/SURFY, differential analysis revealed 264 and 220 proteins significantly (*p* < .05) higher abundance in hTSCs stimulated with EP- or E-sEVs compared to PBS-treated hTSCs (Supplementary Tables S9, S10).

Gene Ontology enrichment analysis (Biological Processes) of proteins distinct between each cluster (EP-sEVs vs. UT; 264 proteins, E-sEVs vs. UT; 220 proteins, and EP-sEVs vs. E-sEVs; 185 proteins; Supplementary Table S10) identified significantly (*p* < .05) enriched terms such as “wound healing,” “cell-substrate adhesion,” and “substrate adhesion-dependent cell spreading” for both EP-sEV and E-sEVs (vs. UT) groups (Figure 2C). For EP-sEV treatment (vs. E-sEVs) processes significantly enriched include “mRNA processing”; *p* < 3.21E-05, and “extracellular matrix organization”; *p* < 4.92E-05” (Figure 2C). For EP-sEV treatment (vs. E-sEVs and UT) specific proteins were shown implicated in regulation of antigen presentation (PSMA5, PSMD11, PSMA2), crosslinking of collagen fibrils (BMP1, LOXL1), NOTCH and VEGF signalling (NOTCH2, PSMD11, PSMA2/NCKAP1, NRAS, ROCK2, PXN), and EPHB-mediated forward signalling (ROCK2, ARPC3, EPHB4), important processes regulating cell invasion (Supplementary Table S9/11). These findings suggest EP-sEVs promote remodeling of cell surface proteome in hTSCs, with changes in proteome landscape associated with regulators of implantation and cell invasion (Table 2).

**TABLE 2:** Cell surfaceome analysis of EP-sEV treatment reveals key biological processes reprogrammed in human trophectoderm cells.

Terms^a^	Description	*p* value	Gene ID
GO:0042060	wound healing	6.28E-09	ADAM17, AK3, AP3B1, CASK, CD9, CNN2, CSRP1, DAG1, DCBLD2, EPHB2, ERBB2, FERMT2, FGA, FGB, FGG, FZD7, GAS6, HBB, NOTCH2, PAPSS2, PRCP, S100A8, STXBP3, TGFBR1
GO:0034446	substrate adhesion-dependent cell spreading	3.08E-07	CRKL, DNM2, FERMT2, FGA, FGB, FGG, FZD7, LAMA5, NRP1, PXN, TEK
GO:0031589	cell-substrate adhesion	3.08E-06	BCAS3, CASK, CRKL, CTNNB1, DAG1, DNM2, FERMT2, FGA, FGB, FGG, FZD7, GAS6, LAMA5, NRP1, PTPRK, PXN, ROCK2, TEK
GO:2001234	negative regulation of apoptotic signaling pathway	1.85E-05	CTNNB1, DNAJA1, FGA, FGB, FGG, LGALS3, NRP1, PARK7, QARS1, RRM2B, TGFBR1, TRAP1, TXNDC12
GO:0007160	cell-matrix adhesion	2.32E-05	BCAS3, CASK, CTNNB1, DAG1, FERMT2, FGA, FGB, FGG, NRP1, PTPRK, PXN, ROCK2, TEK
GO:0006520	cellular amino acid metabolic process	4.21E-05	BLMH, CARS1, CTPS1, FAH, GGT1, GOT1, MARS1, MCCC1, MCCC2, PARK7, QARS1, SARS1, SLC25A13, WARS1
GO:0044089	positive regulation of cellular component biogenesis	5.59E-05	ACTR3, ARL3, BCAS3, DAG1, DNM2, EPHB2, FERMT2, IL1RAP, LCP1, LGALS3, NCKAP1, NRP1, PARK7, PSMC6, PXN, ROCK2, TEK, TGFBR1, WARS1
GO:0051223	regulation of protein transport	5.74E-05	BCAS3, CD200, DNAJA1, ERBB2, FGA, FGB, FGG, GAS6, HADH, IPO5, LCP1, PAM, PARK7, PFKL, SAE1, SCFD1, TM9SF4, TXN, WLS
GO:0050817	coagulation	7.11E-05	AK3, AP3B1, CD9, CSRP1, EPHB2, FGA, FGB, FGG, GAS6, HBB, PAPSS2, STXBP3
GO:0016485	protein processing	1.41E-04	ADAM17, BMP1, CAST, CYCS, FGA, FGB, FGG, GGT1, HP, NCSTN, SEC11A, STOML2
GO:0015980	energy derivation by oxidation of organic compounds	1.49E-04	ATP5PB, ATP5PD, CYCS, DLAT, FH, GAA, IDH3A, PARK7, PYGB, PYGL, SDHB, SLC25A13, STOML2, TRAP1
GO:1903829	positive regulation of protein localization	1.54E-04	BCAS3, CNPY4, EPHB2, ERBB2, FERMT2, FGA, FGB, FGG, GAS6, GNL3, IPO5, LGALS3, PARK7, ROCK2, SAE1, TM9SF4, WLS
GO:0006091	generation of precursor metabolites and energy	1.72E-04	ALDH1L2, ALDOC, ATP5PB, ATP5PD, CYCS, DLAT, FH, GAA, H6PD, IDH3A, PARK7, PFKL, PYGB, PYGL, SDHB, SLC25A13, STOML2, TRAP1
GO:0052547	regulation of peptidase activity	2.00E-04	A2M, C3, C4A, CAST, CYCS, GAS6, NCSTN, PARK7, PEBP1, PRDX5, PSMB8, RECK, ROCK2, S100A8, SERPINB1, SERPINB12, SERPINB6
GO:0071559	response to transforming growth factor beta	2.46E-04	ADAM17, CRKL, DNM2, FERMT2, FOLR1, IGF1R, LTBP1, PTPRK, PXN, ROCK2, TGFBR1, TGFBR3
GO:1990778	protein localization to cell periphery	2.46E-04	ARL3, ATP1B1, CDH2, CNPY4, DAG1, EHD3, EPHB2, EXOC7, GAS6, LAMA5, LGALS3, NECTIN3, RAB13, ROCK2
GO:0031099	regeneration	2.80E-04	CD9, DAG1, FOLR1, FZD7, GAS6, IGF1R, LCP1, MMP2, PTGFRN, TGFBR3
GO:0001655	urogenital system development	3.83E-04	ARL3, CRKL, CTNNB1, EPHB2, FKBP4, KANK2, LAMA5, MMP2, NOTCH2, NRP1, PSAP, RRM2B, TEK, TGFBR1
GO:0043410	positive regulation of MAPK cascade	4.68E-04	ALOX12B, CDH2, CRKL, CTNNB1, ERBB2, FERMT2, FGA, FGB, FGG, FZD7, GAS6, IGF1R, NOTCH2, NRP1, ROCK2, TEK, TGFBR1
GO:0048545	response to steroid hormone	6.72E-04	A2M, CALM3, CARM1, CBX3, FKBP4, GOT1, IGF1R, IGFBP7, KANK2, PAM, PAPPA, PARK7, SAFB2
GO:0006457	protein folding	7.20E-04	CDC37, DNAJA1, DNAJB11, DNAJC10, DNAJC3, DNAJC5, FKBP11, FKBP4, GRPEL1, TRAP1
GO:0034329	cell junction assembly	7.32E-04	BCAS3, CAPZA1, CD9, CDH2, CRKL, CTNNB1, EPHB2, FERMT2, HEG1, IL1RAP, NRP1, PTPRK, RAB13, ROCK2, TEK
GO:0010720	positive regulation of cell development	8.63E-04	CRKL, CTNNB1, DAG1, DNM2, EPHB2, FERMT2, FGA, FGB, FGG, LRP8, NRP1, SRRT
GO:0001667	ameboidal-type cell migration	1.02E-03	ADAM17, BCAS3, CDH2, EPHB4, FOLR1, KANK2, LAMA5, NRP1, PRCP, PTPRG, PXN, RAB13, ROCK2, SPARC, TEK, TGFBR1
GO:0007265	Ras protein signal transduction	1.06E-03	ARHGDIA, CRKL, DNM2, EPHB2, GNB1, HEG1, KANK2, NCKAP1, NOTCH2, NRAS, NRP1, PARK7, ROCK2
GO:0032970	regulation of actin filament-based process	1.12E-03	ARHGDIA, ARPC3, BCAS3, CAPZA1, CNN2, FERMT2, NCKAP1, NOTCH2, NRP1, PAM, PXN, ROCK2, TEK, TGFBR1
GO:0019693	ribose phosphate metabolic process	1.15E-03	AK3, ALDOC, ATP5PB, ATP5PD, CASK, CTPS1, DLAT, HPRT1, MCCC2, PAPSS2, PFKL, PYGL, SLC25A13, STOML2
GO:0009117	nucleotide metabolic process	1.19E-03	AK3, ALDOC, ATP5PB, ATP5PD, CASK, CTPS1, DLAT, HPRT1, MCCC2, PAPSS2, PARK7, PFKL, QPRT, RRM2B, SLC25A13, STOML2
GO:0032956	regulation of actin cytoskeleton organization	1.21E-03	ARHGDIA, ARPC3, BCAS3, CAPZA1, FERMT2, NCKAP1, NOTCH2, NRP1, PAM, PXN, ROCK2, TEK, TGFBR1
GO:0050673	epithelial cell proliferation	1.25E-03	ADAM17, CASK, CTNNB1, ERBB2, FZD7, NCSTN, NOTCH2, NRAS, NRP1, PTPRK, SERPINB1, SPARC, TEK, TGFBR1, TGFBR3
GO:0010631	epithelial cell migration	1.41E-03	ADAM17, BCAS3, EPHB4, KANK2, NRP1, PRCP, PTPRG, PXN, RAB13, ROCK2, SPARC, TEK, TGFBR1
GO:0071692	protein localization to extracellular region	1.44E-03	CD200, FGA, FGB, FGG, HADH, LTBP1, PAM, PARK7, PFKL, RAB13, STEAP3, STXBP3, WLS
GO:0002181	cytoplasmic translation	2.00E-08	AARS1, EIF3A, EIF4H, RPL11, RPL15, RPL21, RPL23, RPL27A, RPL38, RPS14, RPS18, RPS23
GO:0006397	mRNA processing	3.21E-05	ADAR, EFTUD2, HNRNPA0, HNRNPF, HNRNPLL, PRPF40A, PRPF6, RBM25, SAFB2, SART1, SNRPD1, SRRT, SRSF6, SRSF7, U2AF2, VIRMA
GO:0030198	extracellular matrix organization	4.92E-05	BMP1, CCDC80, COL5A2, EMILIN1, GAS6, LOXL1, MMP2, PBXIP1, PTX3, RECK, TGFBR1, THSD4

^a^ Selected terms obtained from cell surfaceome proteomic analysis of hTSCs in response to EP-sEVs, and UT (refer Supplementary Table S10 for complete analysis).

Kinase Enrichment Analysis 3 (KEA3) (Kuleshov et al., 2021) further identified upstream kinases whose substrates from cell surface hTSCs following EP-sEVs treatment were enriched, including cell invasive regulators MAPK3 and AKT1 (Figures 2G–I). We further identify specific growth factor and kinase receptors, including EPHB4/B2, ERBB2, STRAP, EGFR, and PDGFRA, and upregulated expression of pro-invasive regulators associated with focal adhesions (NRP1, PTPRK, ROCK2, TEK) and embryo implantation (FBLN1, NIBAN2, BSG) (Supplementary Table S9) in response to EP-sEV treatment on hTSC surfaceome remodelling. Of note, we report “positive regulation of MAPK cascade”; *p* < 4.68E-04 (Table 2). This highlights proteome reprogramming of trophoblasts by sEVs towards an invasive phenotype.

### Endometrial small extracellular vesicles promote trophectoderm cell invasion *via* MAPK pathway

**FIGURE 3:**
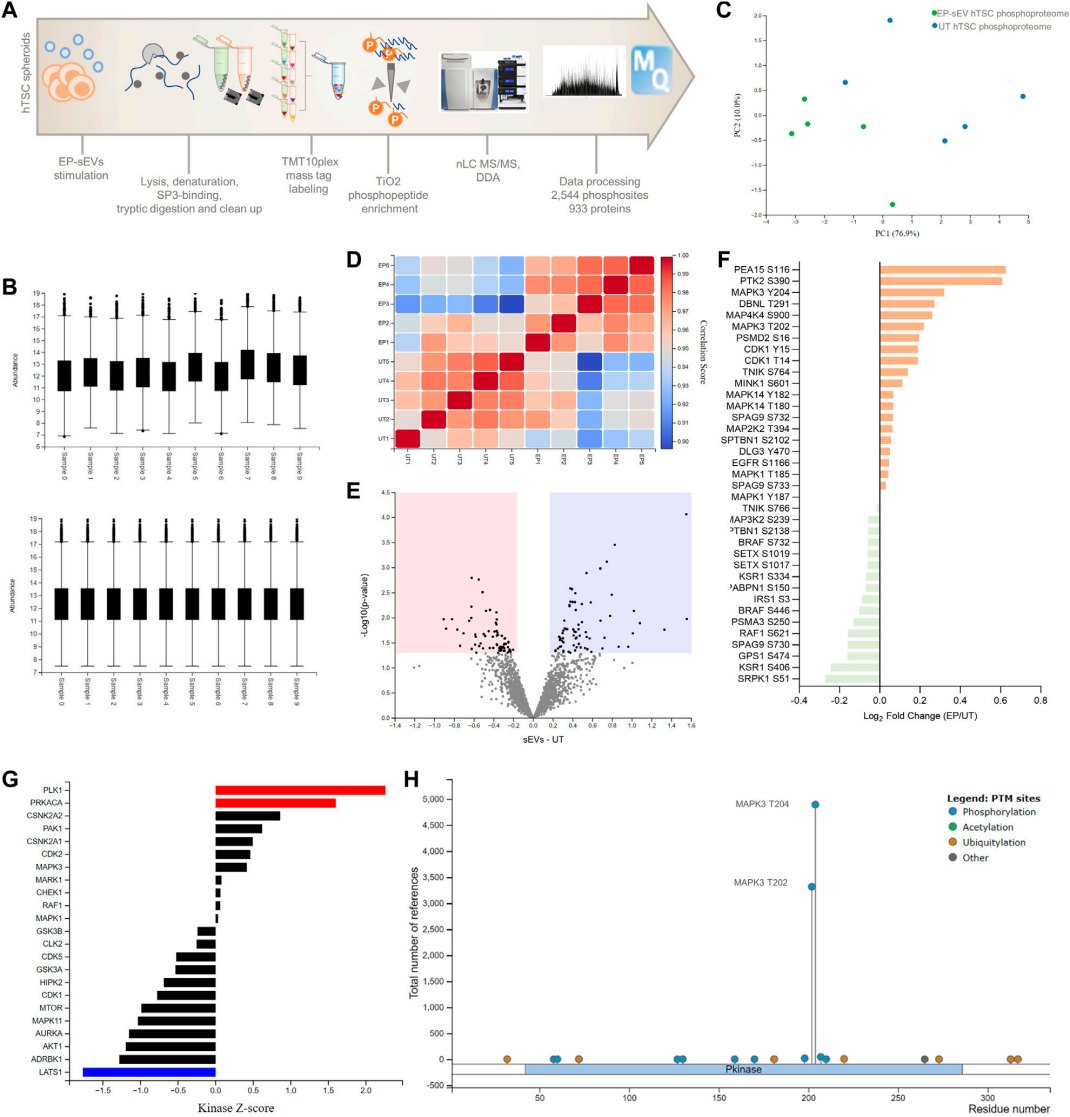
Phosphoproteomic analysis reveal endometrial small extracellular vesicles activate MAPK3 signaling in hTSCs. **(A)** Experimental design of phosphoproteome analysis of hTSCs in response to EP-sEVs. **(B)** Relative abundance of the quantified phosphoproteome before and after z-score normalization [phosphomatics (Leeming et al., 2021)]. Boxes capture lower quartile and upper quartile with median displayed; whiskers min/max. **(C)** Principal component analysis of the phosphoproteome. **(D)** Correlation matrix of the phosphoproteome. **(E)** Volcano plot showing differentially regulated phosphoproteome in hTSCs with EP-sEV treatment. Significantly regulated phosphosites (by t-test from Perseus) were indicated (adjusted *p* < .05 and fold change log_2_ > 0.6). **(F)** Log_2_ (intensity) of specific kinase phosphosites differentially regulated in response to EP-sEV treatment. **(G)** Upstream kinases and/or phosphatases from phosphoproteome profiles in response to EP-sEV treatment were analysed. **(H)** PhosphoSitePlus report on the number of studies for MAPK3 T202 and T204 phosphosites.

**FIGURE 4:**
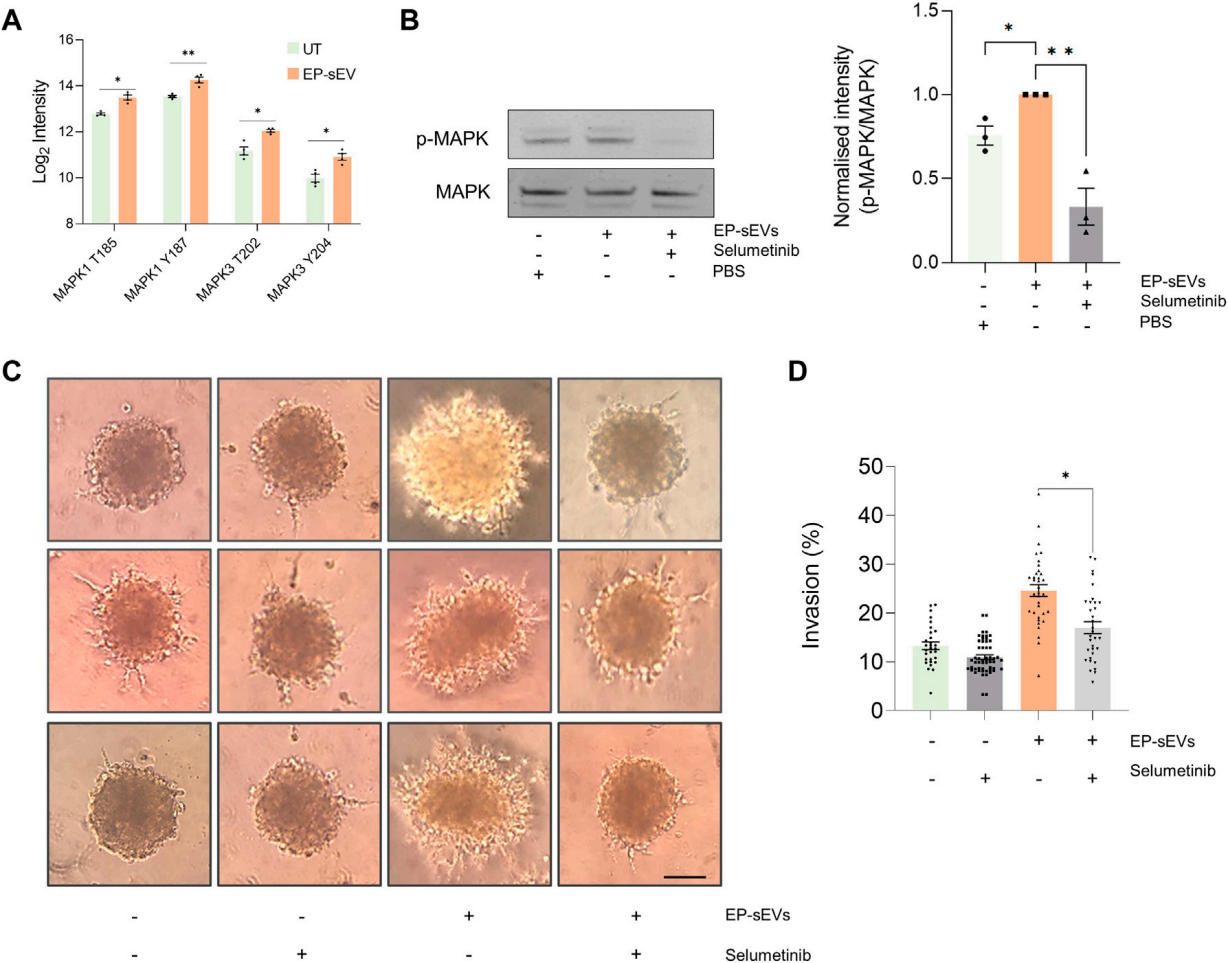
Endometrial small extracellular vesicles promote trophoblast invasion by activating MAPK pathway. **(A)** Phosphopeptide-based quantification of MAPK activation and signaling (mass spectrometry at residue sites MAPK1: T185, Y187, and MAPK3: T202 and T204 in response to EP-sEV treatment based on Student’s T. test (*p < .05, **p < .01). **(B)** Western blot validation of phosphorylated MAPK (ERK1/2) and total MAPK of hTSCs in presence/absence of EP-sEVs and MEK1/2 inhibitor, selumetinib, based on Student’s T. test (*p < .05, **p < .01). Data presented as mean ± SEM (*n* = 3 replicates). **(C)** Cell invasion was assessed by Matrigel™ invasion assay using hTSC spheroids treated with EP-sEVs in presence/absence of selumetinib. Representative images are provided, with data **(D)** presented as mean invasive outgrowth ±SEM (*n* = 7–13 replicates with four invasive fronts measured per spheroid, *p < .0001).

The functional response in target cells is dictated by various signaling pathways activated by EVs, which can be identified by studying changes in the phosphoproteome landscape of target cells. Indeed, interrogation of dysregulated proteins in EP-sEVs-treated hTSCs (global or surface proteomes) identified MAPK3 as upstream kinase implicated in proteome reprogramming (Figures 2D, G). Because of the central role MAPK3 plays in MAPK activation in cellular invasion, we investigated whether sEVs activate MAPK pathway in hTSCs. Thus, we performed phosphoproteome analysis of hTSCs treated with EP-sEVs (compared to untreated hTSCs). We identified 2,543 phosphosites (localization probability >0.75) and 933 phosphoproteins across treatment groups (*n* = 4 per group) (Figures 3A–D, Supplementary Table S12). Of these, 1,252 phosphosites corresponding to 585 proteins were significantly enriched in EP-sEV treated hTSCs compared to untreated hTSCs (Figure 3E, Supplementary Table S12). Their functional enrichment analysis highlighted their involvement in “activation of MAPK signaling, *p* < 2.34E-02,” “regulation of stress-activated MAPK cascade, *p* < 1.70E-03,” “protein phosphorylation, *p* < 4.11E-03” and “ERK1 and ERK2 cascade, *p* < 4.17E-02,” as well as upstream regulators of MAPK signaling, including MAPK3, and various cyclin-dependent and serine/threonine-protein kinase regulators based on kinase Z-score (Figure 3G, Supplementary Table S13). At a phosphosite level, we pinpoint MAPK3/ERK1 T202 and Y204 and MAPK1/ERK2 T185 and Y187 as significantly higher abundance peptides in hTSCs treated with EP-sEVs compared to untreated hTSCs (Figure 3F, H, 4A, Supplementary Table S12).

We demonstrate EP-sEV-mediated significant activation of MAPK by Western blotting using site-specific antibody (*p* < .05) (Figure 4B). Further, to ascertain their direct role in hTSC invasion, we stimulated hTSC spheroids with EP-sEVs that was challenged with selumetinib, a pharmacological inhibitor of MAPK3/ERK1 T202 and Y204 and MAPK1/ERK2 T185 and Y187 phosphosites (Figure 4B). We found that selumetinib significantly attenuated MAPK activation in hTSC cells (*p* < .01) and also abrogated hTSC spheroid invasion into Matrigel matrix to basal levels (*p* < .0001) (Figures 4C, D). Thus, our data show that endometrial sEVs promote hTSC invasion through MAPK activation.

## Discussion

Perturbations in the physiological process of trophoblast cell invasion have been associated with many adverse pregnancy outcomes, such as preeclampsia, intrauterine growth restriction and placenta accreta (Kaufmann et al., 2003; Dey et al., 2004; Tantbirojn et al., 2008; Lyall et al., 2013; Jarvis, 2016; Kim and Kim, 2017; Melchiorre et al., 2022), which together are responsible for over 15 million premature deliveries worldwide every year (World Health Organization and Global, 2015). There is accumulating evidence that trophectoderm/trophoblast invasion is influenced by various types of maternal uterine cells including endothelial cells (Park et al., 2022). Following apposition and adhesion of embryo to the endometrial epithelium, trophoblasts invade into the underlying endometrial stromal decidua to facilitate endometrial and vascular remodeling required to support foetal development (Cakmak and Taylor, 2011; Tang et al., 2018). However, the molecular mechanisms that underpin the critical phase of trophoblast cell invasion remain unclear (Macklon et al., 2002; Park et al., 2022). This study reveals a previously unrecognized but essential step in how small extracellular vesicles (sEVs) from hormonally primed endometrial epithelial cells act as paracrine signalling factors to activate MAPK signaling in trophectoderm cells to facilitate this invasion. Our multi-level quantitative proteomic analysis demonstrates the capacity for hormonally regulated endometrial-derived sEVs to coordinate cellular and cell-surface reprogramming of hTSCs towards a pro-invasive phenotype. This dialogue, regulating dynamics of global and phosphorylation signaling network as well as cell surface proteome of hTSCs, functionally supports trophoblast invasion critical for implantation, cell-matrix adhesion, cell development, protein localization, and cellular remodeling (Table 2).

Phosphorylation regulation is a dynamic and critical signalling regulator of the maternal-embryo interface, immunoregulation and implantation (Ran et al., 2017; Liu et al., 2019). Indeed, various kinase receptor-coupled cytokines and growth factors, such as leukemia inhibitory factor (Stewart et al., 1992; White et al., 2007), interleukin 11 (Robb et al., 1998), and EGF family growth factors (Xie et al., 2007; Large et al., 2014), are essential for normal embryo implantation. Further, phosphorylation of phospholipase C-γ1 (PLCG1) has been reported to support embryo proliferation and development (Ji et al., 1997), in addition to activation of bone morphogenetic protein (BMP) signalling *via* BMPR2 to promote human trophoblast invasion (Zhao et al., 2018). The involvement of soluble secreted factor HGF in regulating this phospho-landscape, mainly MAPK signalling in HTR-8/SVneo trophoblastic cells (Chaudhary et al., 2019), to promote migration (*via* integrins) that is pre-requisite for invasion was also recently reported. The study highlight the dysregulated expression of integrins *via* MAPK pathway during migration, which we also show in our data; an observation that has been long reported (Damsky et al., 1994). Involvement of MAPK signaling in hTSCs invasion and migration is also substantiated by others (Qiu et al., 2004; Lam et al., 2011). Tan et al. (2021), have demonstrated endometrial sEV transfer of miR-100-5p—an upstream regulator of FAK and JNK phosphorylation–to trophoblast cells, activated p-FAK and p-JNK that enhanced trophoblast migration, invasion and proliferation.

Following EP-sEV-treatment, we also identified a major family of cell adhesion molecules important for trophoblast migration, which mediate cell interaction with the extracellular matrix (ECM) (Horton et al., 2016), namely ITGA1/5/V/B3. While such components including ITGAV are enriched in EP-sEVs (Table 1); suggests that sEVs may transfer integrins to modulate target cell surface expression (Park et al., 2019)—whether such targets (on sEVs or following their transfer to target cells) implicate trophectoderm cell interaction [e.g., ITGAV and B3 (Altei et al., 2020)], delivery/cell uptake (Chen and Brigstock, 2016; Li et al., 2021) or tissue-specific homing (Park et al., 2019) remain to be investigated. Moreover, ITGAV forms part of the integrin αVβ3 receptor expressed maternally and on embryo to mediate successful implantation (Illera et al., 2000; Segura-Benitez et al., 2022). In addition to these invasion-associated surfaceome components, SERPINE1 is also expressed in extravillous trophoblasts (EVTs)—a highly invasive trophoblast lineage (Bilban et al., 2010). Moreover, SERPINE1 is expressed by decidual cells and is an important regulator of trophoblast invasion (Kim and Kim, 2017). Of note, tumor and trophoblast invasion share common characteristics in protein expression and mechanism of action, with tumor-derived sEVs shown to directly modify recipient cell motility and invasive capacity through changes in adhesion assembly and ECM components (Sung et al., 2015). Remodelling of trophectoderm cell surface proteins by EP-sEVs also highlight the potential role sEVs play in embryo attachment, since trophectodermal cells are the first point of contact to the maternal endometrial epithelium during implantation (Dey et al., 2004; Evans et al., 2020b). Importantly, MAPK activation and signaling has been shown to coordinate cell surfaceome remodeling (Martinko et al., 2018).

An important future investigation should interrogate the sEV cargo that activates the MAPK pathway. Indeed, sEV could potentially transfer molecular regulators to trophectoderm cells, to activated downstream signaling networks; sEVs were shown to activate p-FAK and p-JNK to enhance trophoblast invasion (Tan et al., 2021). Interestingly, we found that EP-sEVs carry abundant integrins, whether sEVs transfer these integrins to trophectoderm where they interact with EGFR (whose expression was elevated following sEV-treatment), a known activator of Ras-Raf-MAPK/ERK1/2 signal transduction (Pinilla-Macua et al., 2017), warrants investigation. Indeed, how other factors in sEVs which can stimulate phosphorylation of MAPK or other phosphorylation regulators such as PTK2 identified in this study, and their impact on functions associated with adhesion, cell-to-cell contact, and tissue differentiation, does require further investigation.

Overall, this work presents a novel insight into EV-based signalling regulating hTSC invasion. The unique characteristics of EVs to protect their cargo from extracellular degradation and transfer to local (Peinado et al., 2012) or distant sites (Xu et al., 2018), make them promising vehicles for delivery of bioactive therapeutic cargo (Simon et al., 2018; Claridge et al., 2021a) including pro-implantation factors. Insights from our study provide a deeper understanding of endometrial-trophectoderm communication *via* sEVs that can be used to develop a targeted EV delivery approach of therapeutics to modulate implantation. Such advancements highlight the clinical utility of EVs to facilitate implantation (associated with infertility) or alternatively to impede implantation (non-hormonal contraceptive).

## Experimental procedures

### Endometrial cell culture and hormonal treatment

Human Ishikawa endometrial epithelial cells (Nishida et al., 1985) were cultured and maintained in a 1:1 mix of Dulbecco’s Modified Eagle’s Medium/Nutrient Mixture F-12 (DMEM/F-12) (Invitrogen-Gibco, Carlsbad, United States) supplemented with 1% (v/v) Penicillin-Streptomycin (Pen/Strep) (Life Technologies) and 5% (v/v) Fetal Bovine Serum (FBS) (Life Technologies). Cells were maintained at 37°C, 5% CO_2_ and routinely passaged using 0.5% Trypsin-EDTA (Invitrogen-Gibco). For generation of endometrial sEVs, Ishikawa cells were cultured in CELLine™ AD-1000 Bioreactor Classic Flasks (Integra Biosciences) as previously described (Ji et al., 2014). Briefly, Ishikawa cells (∼20 × 10^6^) were seeded into the cultivation chamber, with 500 ml of FBS-supplemented DMEM/F-12 added to the nutrient supply chamber. Cells were cultured for 5 days at 37°C, 5% CO_2_ to expand. Thereafter, cells in the cultivation chamber were gently washed to remove FBS, and media replaced with DMEM/F-12 supplemented with 0.6% (v/v) Insulin-Transferrin-Selenium solution (ITS) (Invitrogen-Gibco) and 1% (v/v) Pen/Strep. Culture media in nutrient supply chamber was replaced weekly, while media in cultivation chambers were collected and replaced according to a cyclic hormonal treatment regime to recapitulate proliferative and secretory phases of the menstrual cycle (Greening et al., 2016).

Ishikawa cells were treated with β-oestradiol (E) (10^−8^ M, E8875; Sigma-Aldrich) supplemented in DMEM/F-12 serum-free media (E-media), and cultured at 37°C, 5% CO_2_ for 48 h to mimic the proliferative phase. E-primed Ishikawa conditioned media (CM) was collected and cells were subsequently treated with β-estradiol (10^−8^ M) and medroxyprogesterone 17-acetate (10^−7^ M, M1629; Sigma-Aldrich) (EP) supplemented in DMEM/F-12 serum-free media (EP-media), and cultured at 37°C, 5% CO_2_ for 48 h to mimic the secretory phase as described (Greening et al., 2016; Poh et al., 2021). The ECC1 human endometrial luminal epithelial cell line was fully validated and cultured as previously described (Greening et al., 2016) with experimental details as for the Ishikawa cells.

Ishikawa endometrial epithelial cells were obtained from Professor Masato Nishida (*via* Prof. Guiying Nie with permission). ECC1 human endometrial luminal epithelial cells were from Professor Lois Salamonsen [American Type Culture Collection (ATCC)].

### Trophectoderm cell culture and spheroid generation

The human trophectodermal stem cells T3-TSC (hTSC) derived from individual blastomeres of donated human embryos (Zdravkovic et al., 2015) were maintained as described (Poh et al., 2021). Briefly, cells were cultured in DMEM/F-12 supplemented with 1% (v/v) Pen/Strep, 10% (v/v) FBS, 10 nM fibroblast growth factor (FGF) (bFGF, R&D Systems) and 10 nM SB431542 (#1614, Tocris Bioscience), in flasks pre-coated with 0.5% (w/v) gelatin, and maintained at 37°C, 5% CO_2_.

hTSC spheroids were generated as described (Evans et al., 2019) with modifications. hTSCs (∼1500 cells) in 100 µl of hTSC media were cultured in round bottom ultra-low attachment 96-well plates (Costar) for 72 h at 37°C, 5% CO_2_ (1 spheroid/well). Spheroids were observed using bright field microscopy and deformed spheroids were excluded from functional use.

### Endometrial small extracellular vesicle isolation and purification

E- and EP-primed CM from Ishikawa cells were centrifuged at 500 g, 5 min and 2,000 g, 10 min at 4°C, with supernatant then centrifuged at 10,000 g, 30 min, 4°C (SW28 rotor; Optima L-90K Ultracentrifuge). The resulting supernatant was centrifuged at 100,000 g, 1 h, 4°C to pellet crude sEVs which were washed in 1 ml PBS at 100,000 g, 1 h, 4°C. sEVs from Ishikawa cells were purified using OptiPrep™ density gradient-based separation as described (Rai et al., 2021c). Briefly, 1 ml volumes of 40%, 20%, 10%, and 0.6 ml of 5% iodixanol solution were layered sequentially in a polypropylene tube (11 × 60 mm). Dilutions of iodixanol solution were made in 0.2 M sucrose/1× PBS solution. sEVs (200 µl) were overlaid and centrifuged at 100,000 *g*, 18 h, 4°C (SW60 rotor, Optima L-90K Ultracentrifuge). Twelve 300 µl fractions were collected, diluted in 1 ml PBS and subjected to a wash spin at 100,000 *g*, 1 h, 4°C. Pellets were re-suspended in 20–100 µl PBS; fractions six to eight were pooled (termed purified E-sEVs or EP-sEVs) and stored at −80°C. The density of each fraction was determined using a control OptiPrep™ gradient overlaid with 200 µl PBS; each fraction was diluted 1:10,000 in MilliQ and absorbance measured at 244 nm using Nanodrop 2000c; absorbance of fractions were converted to density as described (Rai et al., 2021c).

sEV particle size was determined using nanoparticle tracking analysis (NTA, NS300 NanoSight system) as described (Rai et al., 2021c). Syringe pump speed was set to 100 camera detection threshold was at least 10 and temperature set to 25°C. Three technical replicates (60 s video) were recorded and analyzed using NTA software (3.1.45).

Experimental parameters are submitted to EV-TRACK knowledgebase (EV-TRACK ID: EV220403) (Consortium et al., 2017).

### Western blotting

Samples were lysed in western blot buffer [4% (w/v) SDS, 20% (v/v) glycerol and 0.01% bromophenol blue, 0.125 M Tris-hydrochloride, pH 6.8] with 100 mM dithiothreitol (DTT, Thermo Fisher Scientific) as described (Rai et al., 2021c). Membranes were incubated with primary mouse or rabbit antibodies against ALIX (1:1,000 dilution) (3A9; Cell Signaling Technology), TSG101 (1:1,000 dilution) (622696; BD Biosciences), GAPDH (1:1,000 dilution) (D4C6R; Cell Signaling Technology), estrogen receptor alpha (ERα) (1:500 dilution) (D6R2W; Cell Signaling Technology), progesterone receptor A/B (PR A/B) (1:500 dilution) (D8Q2J; Cell Signaling Technology), p-MAPK (9101; Cell signaling), MAPK (9102; Cell signaling), in Tween-PBS (TPBS), overnight at 4°C. Membranes were rinsed with TPBS and incubated with secondary antibodies (1:15,000); IRDye 800CW goat anti-mouse antibody or IRDye 680RD goat anti-rabbit antibody (Li-COR Biosciences), for 1 h while shaking at RT. Membranes were rinsed with TPBS and imaged using Odyssey Infrared Imaging System (Li-COR Biosciences, Nebraska United States), measuring 700 nm and 800 nm wavelengths. Protein-based densitometry to quantify p-MAPK/MAPK relative expression was performed using ImageJ (v1.53c).

### Lipophilic dye labelling of small extracellular vesicles and cell uptake assay

E- and EP-sEVs (10 µg) were incubated with 1 µM DiI (Invitrogen) staining solution at RT, 10 min sEVs (and PBS dye control) were overlaid on a 100 µl cushion of 10% OptiPrep™ (in PBS) and centrifuged at 100,000 *g*, 1 h, 4°C. Supernatants were removed and pellets resuspended in PBS. hTSCs were cultured to ∼70% confluency as droplets in 8-well glass chamber slides (Sarstedt) pre-coated with 0.5% (w/v) gelatin, at 37°C, 5% CO_2_. Cells were incubated with 100 μg/ml E- or EP-sEVs (or PBS dye control) at 37°C, 2 h. Cells were washed in DMEM/F-12 media. Nuclei were stained with Hoechst 33,342 stain (Thermo Fisher Scientific) (10 μg/ml) for 10 min, washed with DMEM/F-12 and fixed with 4% formaldehyde at RT, prior to imaging by Nikon A1R confocal microscope equipped with resonant scanner, using a Plan Fluor 20× MImm (DIC N2, Nikon, Tokyo, Japan). Images were sequentially acquired and are representative of three biological replicates.

### hTSC spheroid invasion assay into Matrigel™ matrix

Transwell-Matrigel™ invasion assays were performed using growth factor-reduced Matrigel™ matrix (Corning), as previously described (Rai et al., 2019). Briefly, 8-well microscopy chambers (Corning) coated with Matrigel™ were overlaid with hTSC spheroids (harvested after 72 h growth, ∼30 spheroids/tube) that were pre-treated with sEVs or PBS alone for 2 h at 37°C. The inserts were nested onto 24-well companion plate (Corning) containing DMEM/F-12 with 1% (v/v) Pen/Strep, 10 nM FGF, 10 nM SB431542 and either E-sEVs or EP-sEVs (50 μg/ml) or PBS alone and incubated for 24 h at 37°C. Subsequently, 50 µl media was removed and mixed 1:1 with Matrigel™ and gently overlaid on spheroids in wells, Matrigel™ allowed to solidify for 30 min at 37°C followed by addition of 200 μl of DMEM/F-12 [10% (v/v) FBS, 1% (v/v) Pen/Strep] to each well. After 48 h, spheroids were imaged using Olympus FSX100 [*n* = 13–20 spheroids analyzed, with four measurements (mm) taken per spheroid]. The extent (%) of invasion was assessed by calculating [(outer diameter—inner diameter)/(inner diameter) × 100] using a digital ruler. Data are presented as average ±SEM. Where indicated, hTSC spheroids were pre-treated with 14 nM Selumetinib (Sellekchem, AZD6244) (Rai et al., 2019) or DMSO for 30 min at 37°C before treatment with EP-sEVs (100 μg/ml) or PBS for 45 min hTSC spheroids were overlaid onto solidified Matrigel™ to invade for 48 h and imaged as described (*n* = 7–13 spheroids analyzed).

### Sample preparation and mass spectrometry of endometrial sEV and cell proteome

Global mass spectrometry-based proteomics of Ishikawa cells or derived sEVs (*n* = 3), or hTSCs following sEV treatment (*n* = 5) (10 μg) was performed as described (Claridge et al., 2021b; Lozano et al., 2022) using single-pot solid-phase-enhanced sample preparation (SP3) method (Hughes et al., 2019). Briefly, Ishikawa cell and sEV derivatives were solubilized in 1% (v/v) SDS, 50 mM tetraethylammonium bromide (TEAB) pH 8.0, while hTSCs were solubilized in 1% (v/v) SDS, 50 mM HEPES pH 8.0. Samples were incubated at 95°C for 5 min, with cell lysates tip probe sonicated (10 s, 23 amplitude) (Misonix—S-4000 Ultrasonic Liquid Processor). Samples were reduced with 10 mM DTT for 50 min at 25°C followed by alkylation with 20 mM iodoacetamide (IAA) for 30 min at 25°C in the dark. The reaction was quenched to a final concentration of 20 mM DTT. Magnetic beads were prepared by mixing SpeedBeads™ magnetic carboxylate modified particles (65152105050250, 45152105050250, Cytiva) at 1:1 (v:v) ratio and washing twice with 200 µl MS-water. Magnetic beads were reconstituted to a final concentration of 100 μg/μl. Magnetic beads were added to the samples at 10:1 beads-to-protein ratio and 100% ethanol (EA043, ChemSupply) added for a final concentration of 50% ethanol (v/v). Protein-bound magnetic beads were washed three times with 200 µl of 80% ethanol and reconstituted in 50 mM TEAB and digested with trypsin (Promega, V5111) at a 1:50 enzyme-to-substrate ratio for 16 h at 37°C with constant shaking (1,000 rpm). The peptide mixture was acidified to a final concentration of 2% formic acid (FA) (pH ∼1–2) and centrifuged at 20,000 *g* for 1 min. The peptide digests were frozen at −80°C and dried by vacuum centrifugation (Savant SPD121P, Thermo Fisher Scientific), reconstituted in 0.07% trifluoroacetic acid (TFA), and quantified by Fluorometric Peptide Assay (23,290, Thermo Fisher Scientific) as per manufacturer’s instruction.

Peptides were analyzed on a Dionex UltiMate NCS-3500RS nanoUHPLC coupled to a Q-Exactive HF-X hybrid quadrupole-Orbitrap mass spectrometer equipped with nanospray ion source in positive mode as described (Greening et al., 2021; Kompa et al., 2021). Peptides were loaded (Acclaim PepMap100 C18 3 μm beads with 100 Å pore-size, Thermo Fisher Scientific) and separated (1.9 µm particle size C18, 0.075 × 250 mm, Nikkyo Technos Co. Ltd.,) with a gradient of 2%–28% acetonitrile containing 0.1% FA over 95 min at 300 nl·min^−1^ followed by 28–80% from 95–98 min at 300 nL·min^−1^ at 55°C (butterfly portfolio heater, Phoenix S&T). An MS1 scan was acquired from 350 to 1,650 m/*z* [60,000 resolution, 3 × 10^6^ automatic gain control (AGC), 128 m injection time] followed by MS/MS data-dependent acquisition (top 25) with collision-induced dissociation and detection in the ion trap (30,000 resolution, 1 × 10^5^ AGC, 60 m injection time, 28% normalized collision energy, 1.3 m/*z* quadrupole isolation width). Unassigned, 1, six to eight precursor ions charge states were rejected and peptide match disabled. Selected sequenced ions were dynamically excluded for 30 s. Data was acquired using Xcalibur software v4.0 (Thermo Fisher Scientific). All MS-based proteomics data (cellular, sEV, phosphoproteome, surfaceome) have been deposited to the ProteomeXchange Consortium *via* the PRIDE partner repository and are available *via* ProteomeXchange with identifier (PXD027642). Therefore, for this study, biological replicates were performed as indicated: global proteome analysis (*n* = 5) and cell surfaceome analysis (*n* = 3).

### Sample preparation and mass spectrometry of hTSC phosphoproteome

hTSCs (∼20,000) were seeded in 96-well plates pre-coated with 0.5% (w/v) gelatin and cultured to 100% confluency. Cells were serum-starved for 24 h prior to treatment with 100 μg/ml purified EP-sEVs or PBS control for 15 min at 37°C, 5% CO_2_ (*n* = 4). Cells were immediately washed 2× in ice-cold PBS and lysed in-well with MS lysis buffer [1% (v/v) SDS, 1:100 HALT protease phosphatase inhibitor cocktail (Thermo Fisher Scientific, 78,442), 50 mM HEPES pH 8] on ice for 5 min, heat treated at 95°C for 5 min prior to SP3 sample preparation. Cell lysates were reduced, alkylated, quenched and trypsin digested as described, with the resulting peptide mixture acidified to a final concentration of 2% FA, and centrifuged at 20,000 *g* for 1 min. For phosphoproteomic analysis by tandem mass tag (TMT) multiplexing, peptides were labelled with 10-plex TMT labels according to the manufacturer’s instructions (Thermo Fisher Scientific, 90406/A34807, lot UG287488/278919). A list of the sample labelling strategy is available in PRIDE proteomeXchange (PXD027642). Peptides were labelled in a final concentration of 50% acetonitrile for 90 min at RT followed by de-acylation with 0.25% hydroxylamine for 15 min at RT and quenching with 0.1% TFA. Peptides from each 10-plex experiment were pooled and digests lyophilised by vacuum centrifugation and reconstituted in Binding/Equilibration Buffer for phosphopeptide enrichment, using the High-Select™ TiO2 Phosphopeptide Enrichment workflow, as per manufacter’s instructions (Thermo Fisher Scientific, A32993). Briefly, TMT-labelled peptide digests were transferred to a pre-equilibrated TiO_2_ spin tip and centrifuged twice at 1,000 *g*, 5 min. The column was washed twice with binding/equilibration buffer and subsequent wash buffer at 3,000 *g*, 2 min, followed by a wash with MS-grade water at 3,000 *g*, 2 min. Phosphopeptides were eluted in 100 µl phosphopeptide elution buffer by centrifugation at 1,000 *g*, 5 min, dried by vacuum centrifugation and reconstituted in 0.07% TFA, before quantification by Colorimetric Peptide Assay (Thermo Fisher Scientific, 23,275) as per manufacturer’s instructions.

MS-based proteomic analysis of TMT-labelled phosphopeptides was performed on a Dionex UltiMate NCS-3500RS nanoUHPLC coupled to a Q-Exactive HF-X hybrid quadrupole-Orbitrap mass spectrometer equipped with nanospray ion source in positive mode. TMT labelled peptides were separated (1.9 µm particle size C18, 0.075 × 250 mm, Nikkyo Technos Co. Ltd.,) with a gradient of 4%–28% ACN containing 0.1% FA over 224 min at 300 nl/min at 55°C (butterfly portfolio heater, Phoenix S&T). The MS1 scan was acquired from 300 to 1650 m/z (60,000 resolution, 3e6 AGC, 128 m injection time) followed by MS/MS data-dependent acquisition of the top 15 ions with HCD (30,000 resolution, 1e5 AGC, 60 ms injection time, 33 NCE, 0.8 m/z isolation width). Unassigned, 1, six to eight precursor ions charge states were rejected and peptide match disabled. Selected sequenced ions were dynamically excluded for 30 s. Data was acquired using Xcalibur software v4.0. Therefore, for this study, biological replicates of the phosphoproteome analysis (*n* = 4).

### Sample preparation and mass spectrometry of hTSC cell surfaceome

hTSCs (∼80,000) were seeded in 24-well plates pre-coated with 0.5% (w/v) gelatin and cultured to ∼80% confluency, serum-starved for 24 h prior to treatment with E- or EP-sEVs (50 μg/ml) or PBS for 48 h (*n* = 3). Cell surface proteins were biotinylated using Pierce™ Cell Surface Biotinylation (A44390, Thermo Fisher Scientific) as described (Rai et al., 2021c). Briefly, cells were washed twice with PBS and labeled with 0.25 mg/ml EZ-Link Sulfo-NHS-SS-Biotin for 10 min at RT. Cells were washed twice with ice-cold Tris-buffered saline and lysed (30 min on ice) as per manufacturer’s instructions. Biotin-labelled proteins were captured onto NeutrAvidin Agarose slurry (30 min at RT with end-over-end mixing on a rotor). Samples were loaded onto epTIPS (Eppendorf, 200 μl) fitted with 20 μm nylon net (NY2004700, Merck Millipore), washed 3× as per manufacturers instruction, and reduced in 10 mM DTT in 100 mM TEAB for 45 min at 25°C. Eluted proteins were alkylated, quenched and trypsin digested as described, with resultant peptides acidified (pH ∼2.0) to a final concentration of 2% FA, peptides desalted using SDB-RPS Stage-Tips (Rai et al., 2021a) followed by elution with 30%–80% acetonitrile, 0.1% TFA, and dried by vacuum centrifugation. Peptides were reconstituted in 0.07% TFA and quantified by Fluorometric Peptide Assay. Peptides were analysed on a Dionex UltiMate NCS-3500RS nanoUHPLC coupled to a Q-Exactive HF-X hybrid quadrupole-Orbitrap mass spectrometer equipped with nanospray ion source in positive data-dependent acquisition mode over 95 min gradient, as described (Rai et al., 2021c).

### Data processing and database analyses

Raw mass spectrometric data were processed in MaxQuant (v1.6.14.0) with its built-in search engine Andromeda (Cox and Mann, 2008) to perform peptide identification and quantification as described (Kompa et al., 2021; Lozano et al., 2022). Database search was with the Andromeda search engine against the Human-only canonical sequence database (2020) with a contaminants database employed. Cysteine carbamidomethylation was set as a fixed modification and acetyl (Protein N-term) and methionine oxidations as variable modifications. Differential search parameters included: TMT tags on peptide N terminus/lysine and carbamidomethylation of cysteine set as a fixed modification; for phosphoproteome analysis phosphorylation of serine, threonine and tyrosine were set as variable modifications; for biotin surface proteome analysis additional Thioacyl (DSP) {[C (3)H (4)OS]} (Rai et al., 2021c) was employed. Enzyme specificity was set as C-terminal to arginine and lysine using trypsin protease, and a maximum of two missed cleavages allowed. Match between runs options were enabled, with the matching time window set to 1 min, with label-free protein quantitation (LFQ) performed where applicable. False discovery rate (FDR) at the PSM, protein, and site levels were each 1%. Peptides were identified with an initial precursor mass deviation of up to 7 ppm and a fragment mass deviation of 20 ppm. A maximum of two missed cleavages were allowed. Protein group or phosphorylation site tables were imported into Perseus (v1.6.7) for analysis, with contaminants and reverse peptides removed.

### Bioinformatic and statistical analyses

Data analysis was performed using Perseus of the MaxQuant computational platform (Tyanova et al., 2016), phosphomatics (Leeming et al., 2021), Kinase Enrichment Analysis 3 (KEA3) (Kuleshov et al., 2021) and R programming language (Notaras et al., 2021a; Notaras et al., 2021b). Protein intensities were log2 transformed and subjected to principal component analysis (PCA) with missing values imputed from normal distribution (width 0.3, downshift 1.8) and Student’s t-test. Phosphosites quantified in at least one sample were log2 transformed, median centred, and scaled within each treatment/condition. Missing values were then imputed for each phosphosite based on its pattern of missingness in the EP-sEV or untreated groups. Specifically, phosphopeptides were quantified in at least two out of three biological replicates in each group. Hierarchical clustering was performed using Euclidian distance and average linkage clustering. The volcano plot of student’s t-test *p*-value *versus* log2 fold change was generated. g: Profiler and Reactome databases were utilized for functional enrichment and network/pathway analysis, significance *p* < .05 as described (Rai et al., 2021c; Notaras et al., 2022). To further annotate function, phosphoproteome profiles were analysed with the PhosphoSitePlus database (Hornbeck et al., 2019). Surfaceome data analyzed includes proteins previously experimentally verified as a cell-surface (CSPA) (Bausch-Fluck et al., 2015) or predicted as surfaceome proteins based on SURFY (Bausch-Fluck et al., 2018). Cytoscape (v3.8.1) (Shannon et al., 2003) was used to generate STRING based protein–protein interaction network analysis. Bar plots were generated using GraphPad Prism (v9.0.0). Statistical analyses were performed using Perseus and GraphPad Prism, with unpaired two-sample Student’s T. test or one-way ANOVA performed (statistical significance defined at *p* < .05).

## Data Availability

The datasets presented in this study can be found in online repositories. The names of the repository/repositories and accession number(s) can be found in the article/Supplementary Material.

## Supplementary material

SUPPLEMENTARY FIGURE S1 Western blot analysis of Ishikawa cells following i*n vitro* hormonal treatment with estrogen (E; β-oestradiol 10^−8^ M) (proliferative phase), and subsequent E and progesterone (P) (EP; β-estradiol 10^−8^ M and medroxyprogesterone 17-acetate 10^−7^ M) (secretory phase); using anti-ERα, -PRA/ -PRB and -GAPDH (load control) (*n* = 3).

SUPPLEMENTARY FIGURE S2 Principal component analysis of endometrial cell and small extracellular vesicle proteome. Principal component analysis of proteins (with their respective intensities) identified in **(A)** Ishikawa whole cell lysate (parental cells) and Ishikawa sEVs (combined E- and EP-sEVs), and **(B)** Ishikawa E- and EP-sEVs (*n* = 3).

SUPPLEMENTARY FIGURE S3 Venn diagram of proteins uniquely identified/ differentially expressed in E- and EP-sEVs (fold change log2 > ±1, Student’s T.test *p* < 0.05, identified in ≥2 biological replicates). **(A)** Gene ontology over-representation analysis (biological processes, *p* < 0.05) and UniProt functional annotation of significantly regulated proteins in sEVs regulated by EP treatment. **(B)** Comparative analysis of significantly regulated proteins in EP-sEVs with known players of implantation (Enciso et al., 2018; Matorras et al., 2018), human uterine fluid secretory phase proteome (Rai et al., 2021a), endometrial, receptivity (Azkargorta et al., 2018; Diaz-Gimeno et al., 2011), and window of implantation (WOI) signature (Altmae et al., 2017).

SUPPLEMENTARY FIGURE S4 Comparative analysis of endometrial cell-derived sEV cargo. Venn diagram of protein identifications from sEVs derived from Ishikawa and ECC1 cell lines (Greening et al., 2016). Commonly identified proteins in **(A)** EP-sEVs are involved in phosphorylation, migration, developmental and invasion-related processes, and players in **(B)** E-sEVs are involved in phosphorylation, invasion and proliferation-related processes. Functional annotations derived from (UniProt, 2019).

SUPPLEMENTARY FIGURE S5 Endometrial small extracellular vesicle taken up by trophectoderm cells. Live fluorescence and bright-field analyses of hTSCs with endometrial-derived E- and EP-sEVs (2 h). Endometrial sEVs are distributed intracellularly (z-stack). Images are representative of individual biological replicates.

SUPPLEMENTARY FIGURE S6 hTSC spheroid Matrigel^TM^ invasion assay. Phase-contrast microscopy of hTSC spheroids Matrigel^TM^ invasion assay treated with **(A)** E-/EP-sEVs (50 µg/ml) or **(B)** EP-sEVs (100 µg/ml) with/without Selumetinib (14 nM), with volume matched untreated (PBS, UT) control. Scale bar, 100 µm.

SUPPLEMENTARY FIGURE S7 Western blot analysis images for **(A)** Ishikawa cells treated with E or EP for 48 h, anti-ERα, -PR A/B and -GAPDH; **(B)** density fractions of endometrial sEVs, anti-ALIX and -TSG101. (**(Ci-iii)**, independent replicates) phospho-MAPK (ERK1/2) and MAPK and/or selumetinib, anti-p-MAPK, -MAPK and -GAPDH; EP-sEV treatment/PBS.
