# Supplementary figures and images for: Endometrial small extracellular vesicles regulate human trophectodermal cell invasion by reprogramming the phosphoproteome landscape
Source: Front Cell Dev Biol. 2022 Dec 22;10:1078096. doi: 10.3389/fcell.2022.1078096 (PMC9813391; doi:10.3389/fcell.2022.1078096)

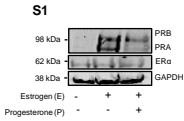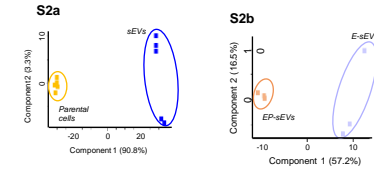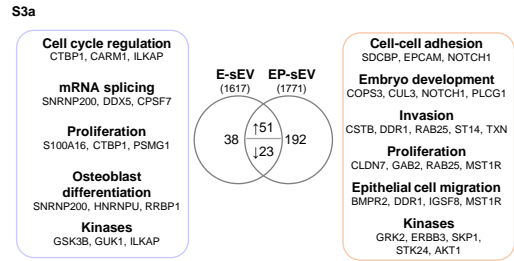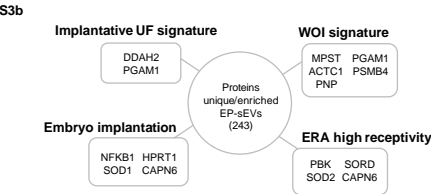

S4a

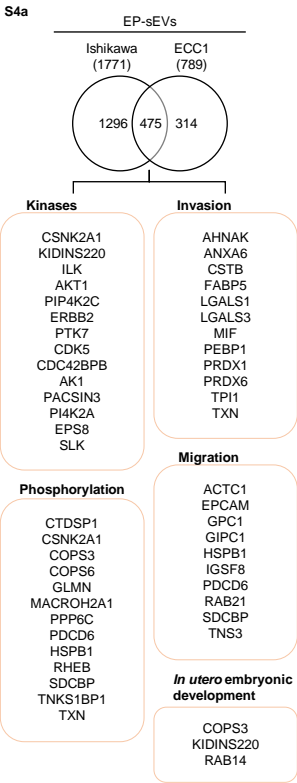

S4b

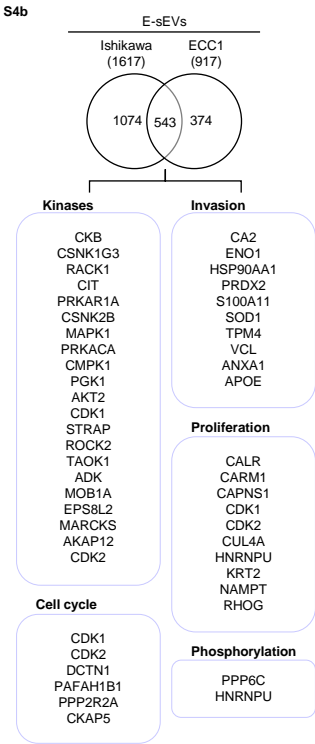

S5

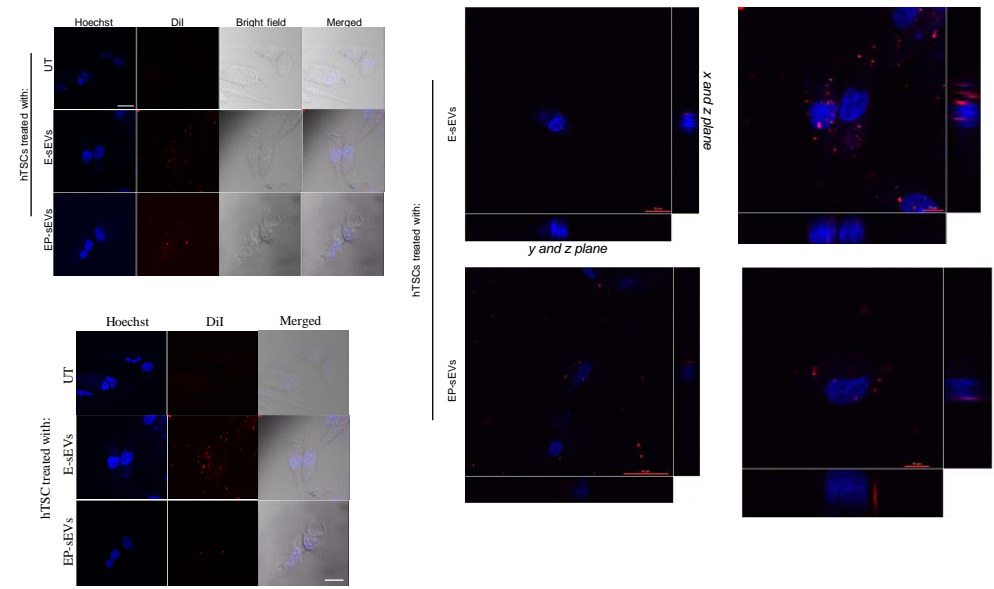

S6

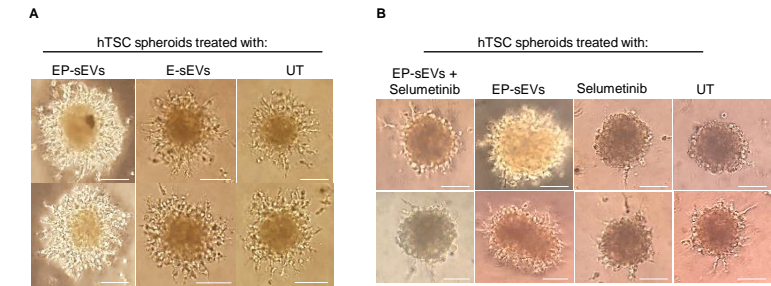

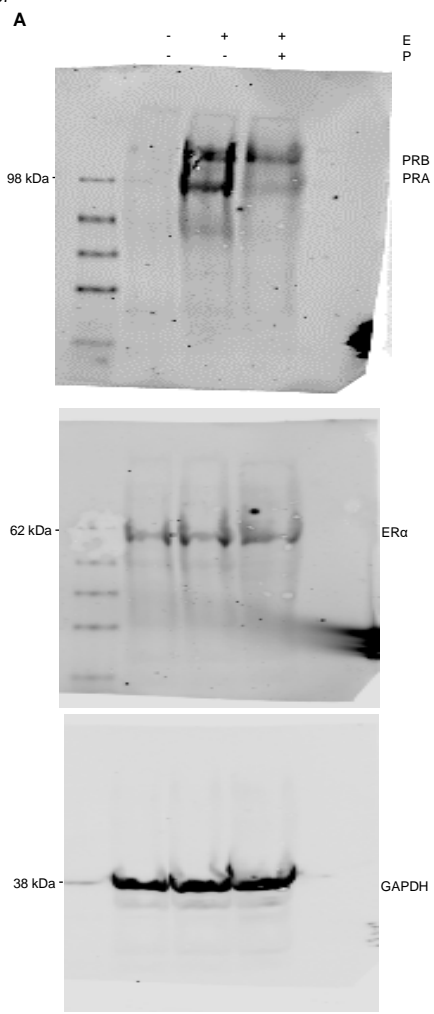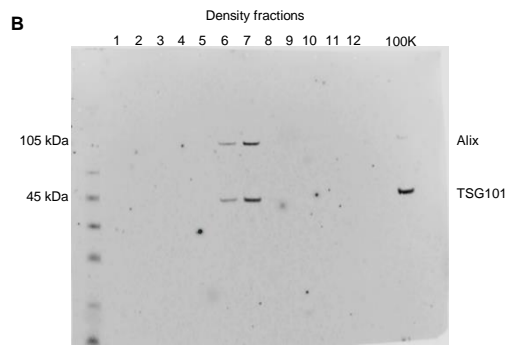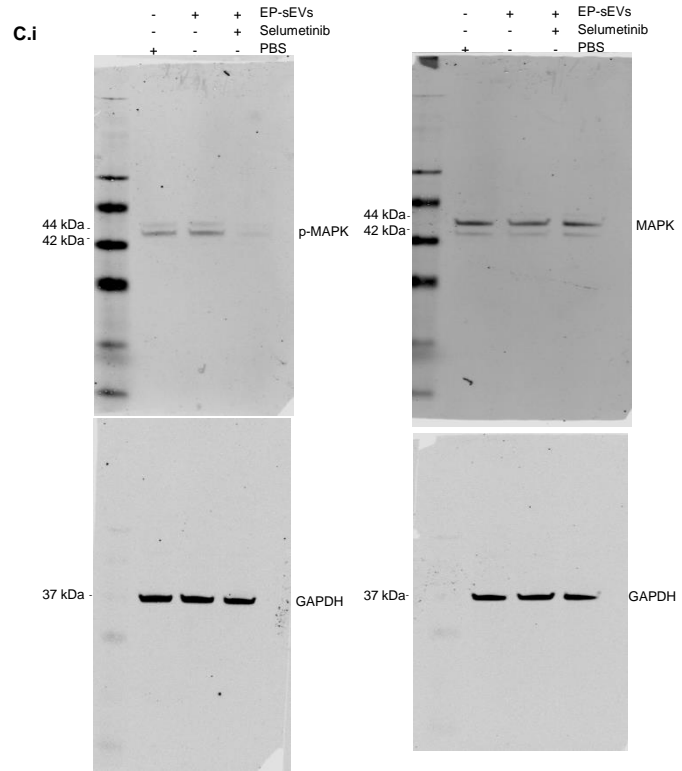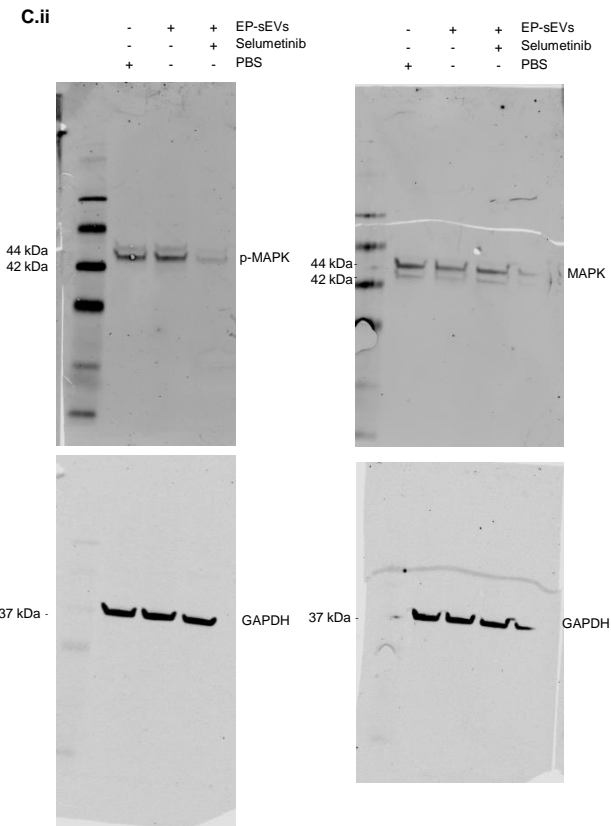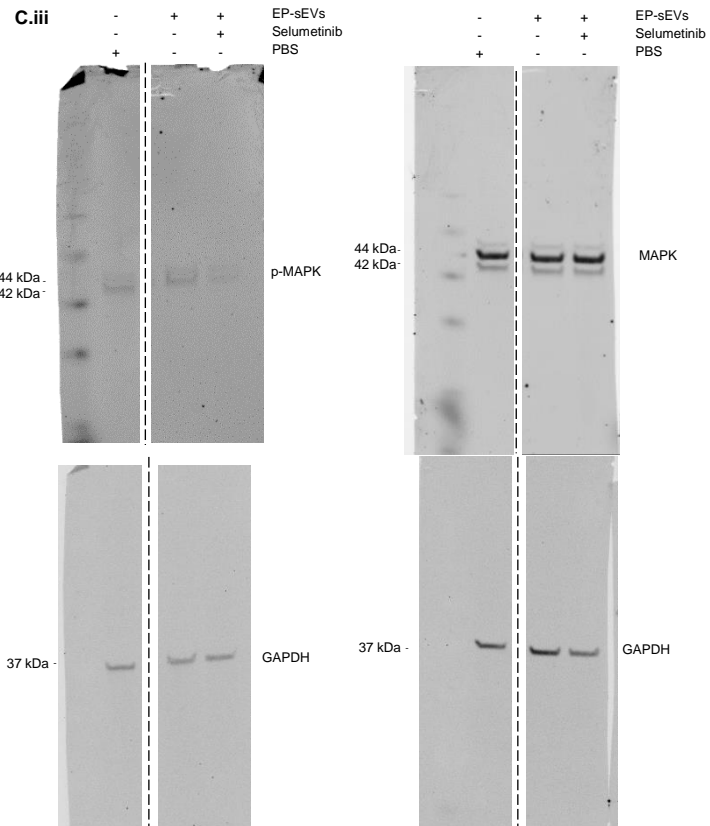

Supplement: Supplementary file 2 [file DataSheet1.PDF]
