# Supplementary material for: Endometrial small extracellular vesicles regulate human trophectodermal cell invasion by reprogramming the phosphoproteome landscape
Source: Front Cell Dev Biol. 2022 Dec 22;10:1078096. doi: 10.3389/fcell.2022.1078096 (PMC9813391; doi:10.3389/fcell.2022.1078096)
Supplement: Supplementary file 3 [file Presentation2.PPTX]

## Slide 1
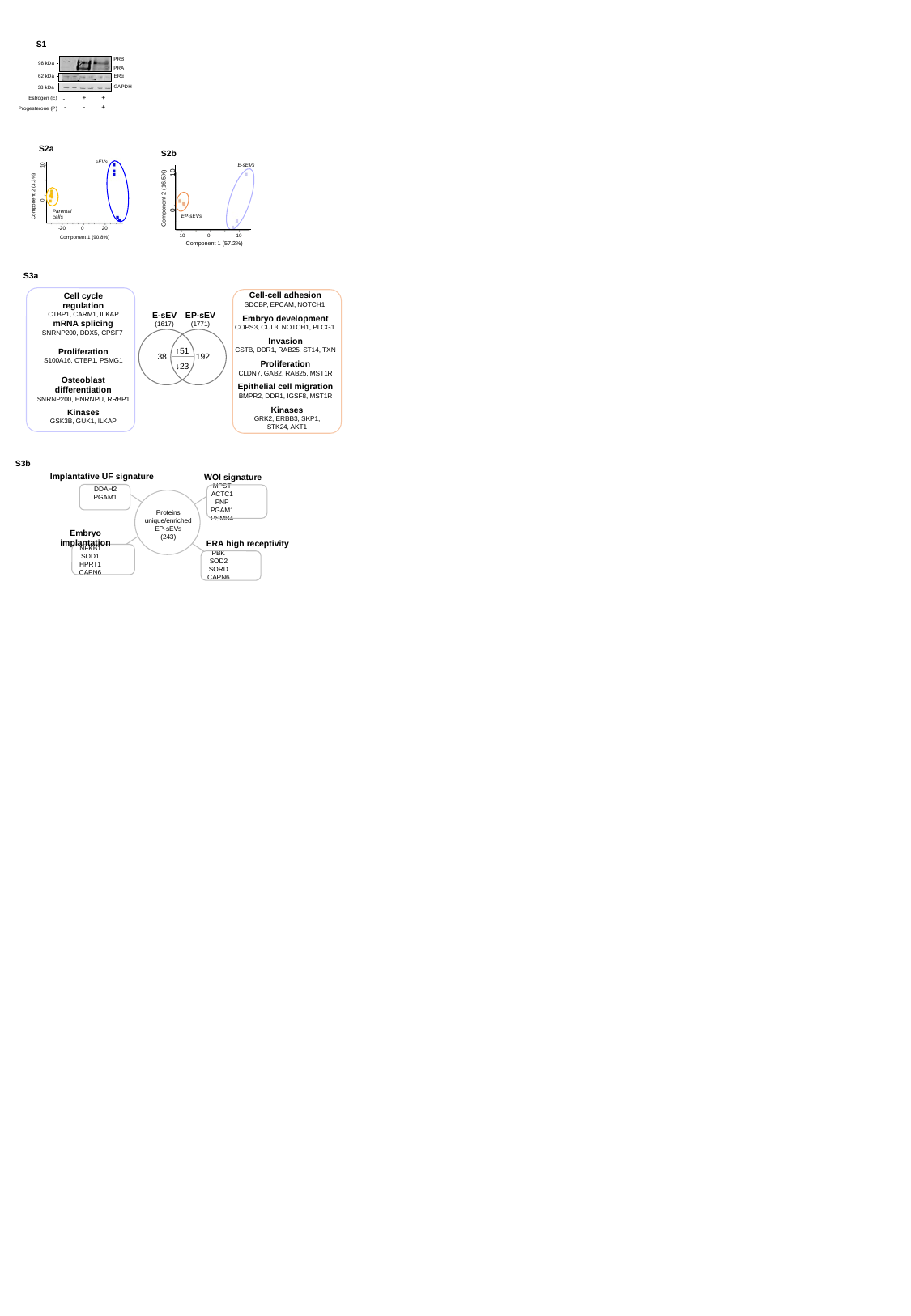

S1
 PRB
98 kDa
 PRA
62 kDa
ERα
GAPDH
38 kDa
+
+
-
-
-
+
Estrogen (E)
Progesterone (P)
S2a
S2b
sEVs
10
Component 2 (3.3%)
0
Parental cells
-20
0
20
Component 1 (90.8%)
E-sEVs
10
Component 2 (16.5%)
0
EP-sEVs
0
10
-10
Component 1 (57.2%)
S3a
Cell cycle regulation
CTBP1, CARM1, ILKAP
Cell-cell adhesion
SDCBP, EPCAM, NOTCH1
E-sEV
(1617)
EP-sEV
(1771)
Embryo development
COPS3, CUL3, NOTCH1, PLCG1
mRNA splicing
SNRNP200, DDX5, CPSF7
38
192
Invasion
CSTB, DDR1, RAB25, ST14, TXN
↑51
↓23
Proliferation
S100A16, CTBP1, PSMG1
Proliferation
CLDN7, GAB2, RAB25, MST1R
Osteoblast differentiation
SNRNP200, HNRNPU, RRBP1
Epithelial cell migration
BMPR2, DDR1, IGSF8, MST1R
Kinases
GRK2, ERBB3, SKP1, STK24, AKT1
Kinases
GSK3B, GUK1, ILKAP
S3b
Implantative UF signature
WOI signature
DDAH2
PGAM1
MPST
ACTC1
PNP
PGAM1
PSMB4
Proteins unique/enriched
EP-sEVs
(243)
Embryo implantation
NFKB1 SOD1
HPRT1
CAPN6
ERA high receptivity
PBK
SOD2
SORD
CAPN6

## Slide 2
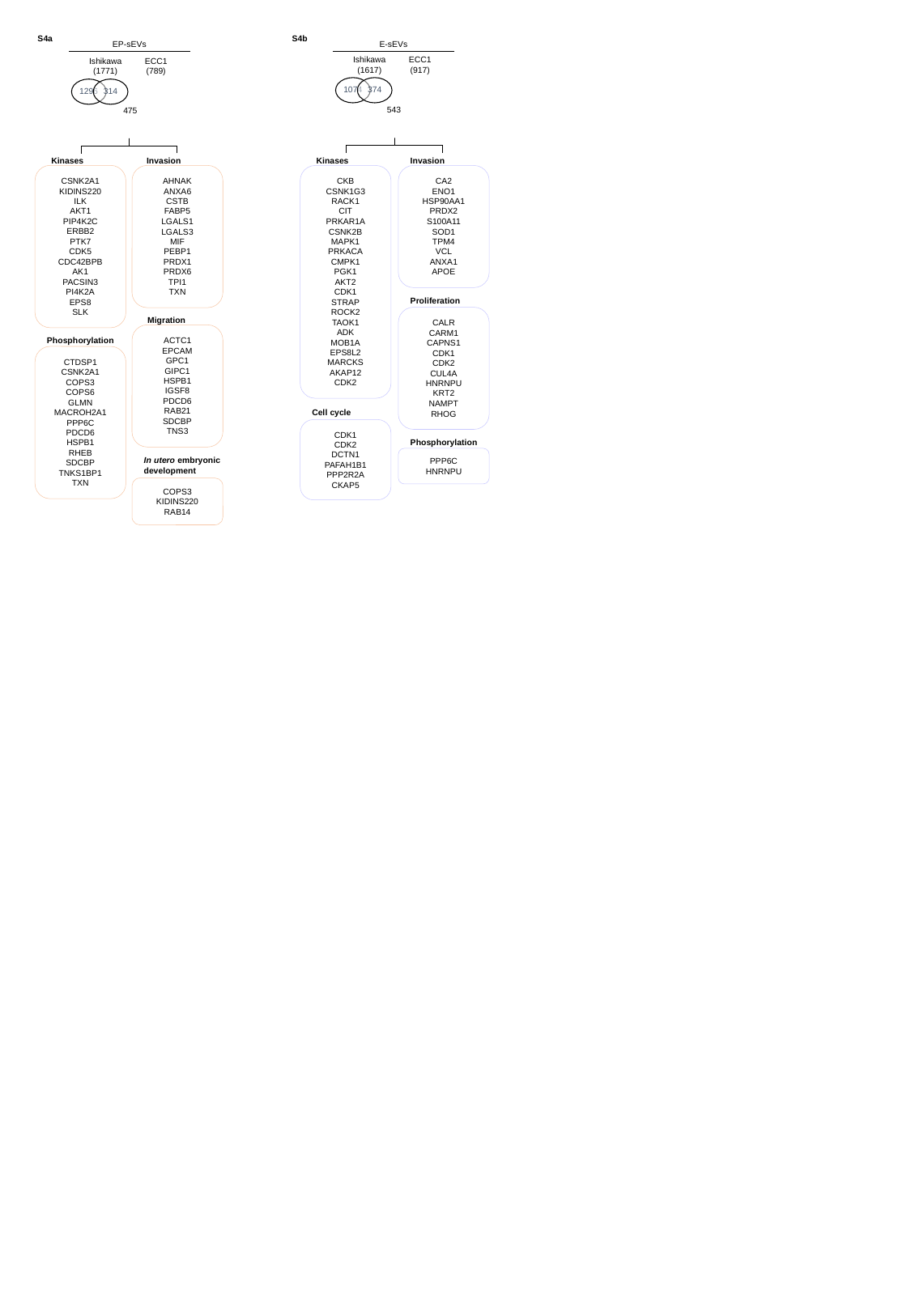

S4a
S4b
E-sEVs
EP-sEVs
Ishikawa
(1617)
ECC1
(917)
543
Ishikawa
(1771)
ECC1
(789)
475
Kinases
Invasion
CSNK2A1
KIDINS220
ILK
AKT1
PIP4K2C
ERBB2
PTK7
CDK5
CDC42BPB
AK1
PACSIN3
PI4K2A
EPS8
SLK
AHNAK
ANXA6
CSTB
FABP5
LGALS1
LGALS3
MIF
PEBP1
PRDX1
PRDX6
TPI1
TXN
Migration
ACTC1
EPCAM
GPC1
GIPC1
HSPB1
IGSF8
PDCD6
RAB21
SDCBP
TNS3
Phosphorylation
CTDSP1
CSNK2A1
COPS3
COPS6
GLMN
MACROH2A1
PPP6C
PDCD6
HSPB1
RHEB
SDCBP
TNKS1BP1
TXN
In utero embryonic development
COPS3
KIDINS220
RAB14
Invasion
Kinases
CKB
CSNK1G3
RACK1
CIT
PRKAR1A
CSNK2B
MAPK1
PRKACA
CMPK1
PGK1
AKT2
CDK1
STRAP
ROCK2
TAOK1
ADK
MOB1A
EPS8L2
MARCKS
AKAP12
CDK2
CA2
ENO1
HSP90AA1
PRDX2
S100A11
SOD1
TPM4
VCL
ANXA1
APOE
Proliferation
CALR
CARM1
CAPNS1
CDK1
CDK2
CUL4A
HNRNPU
KRT2
NAMPT
RHOG
Cell cycle
CDK1
CDK2
DCTN1
PAFAH1B1
PPP2R2A
CKAP5
Phosphorylation
PPP6C
HNRNPU

## Slide 3
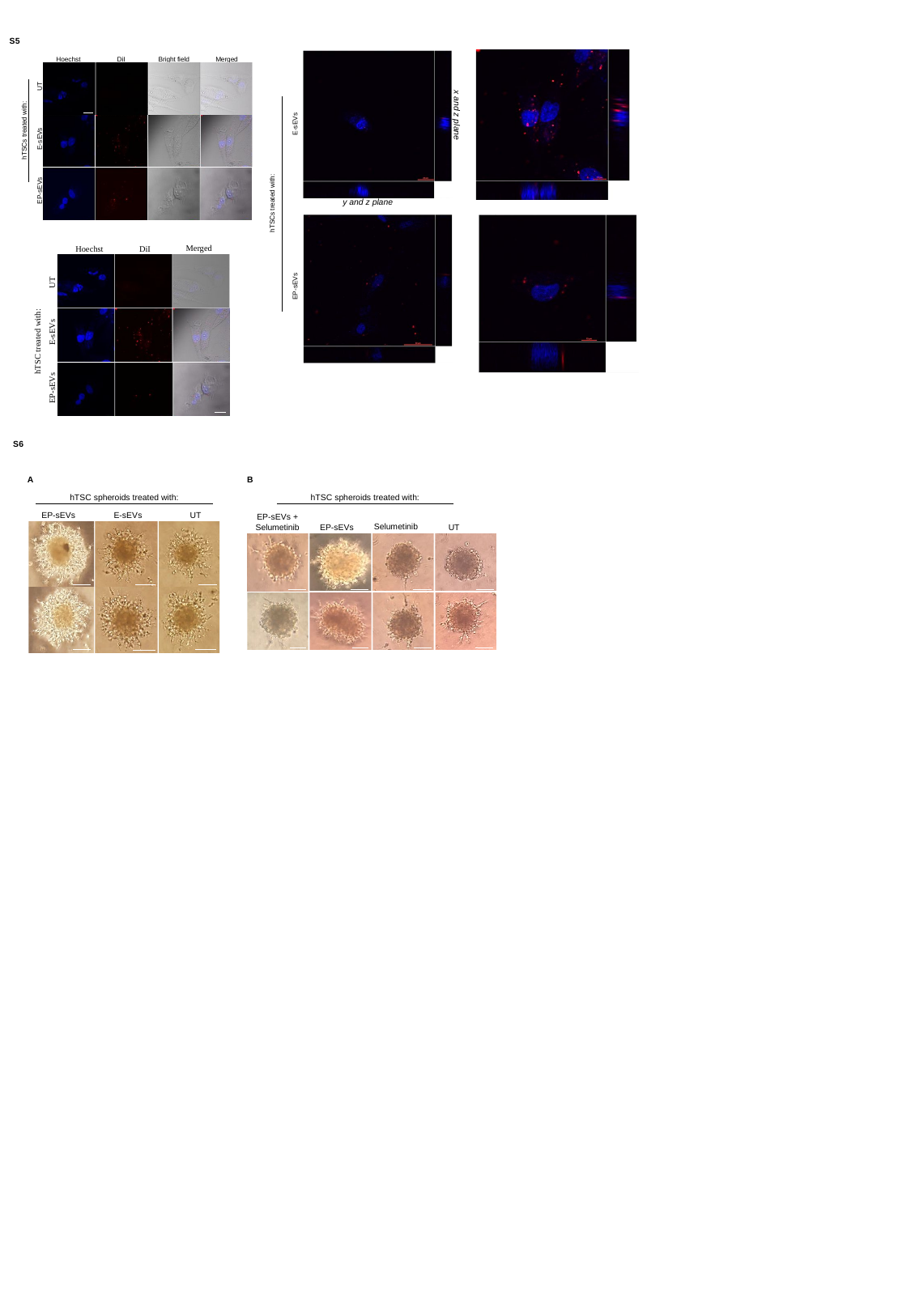

S5
Hoechst
DiI
Bright field
Merged
UT
hTSCs treated with:
E-sEVs
EP-sEVs
x and z plane
E-sEVs
y and z plane
hTSCs treated with:
Merged
Hoechst
DiI
UT
E-sEVs
hTSC treated with:
EP-sEVs
EP-sEVs
S6
A
B
hTSC spheroids treated with:
hTSC spheroids treated with:
EP-sEVs
UT
E-sEVs
EP-sEVs + Selumetinib
Selumetinib
EP-sEVs
UT

## Slide 4
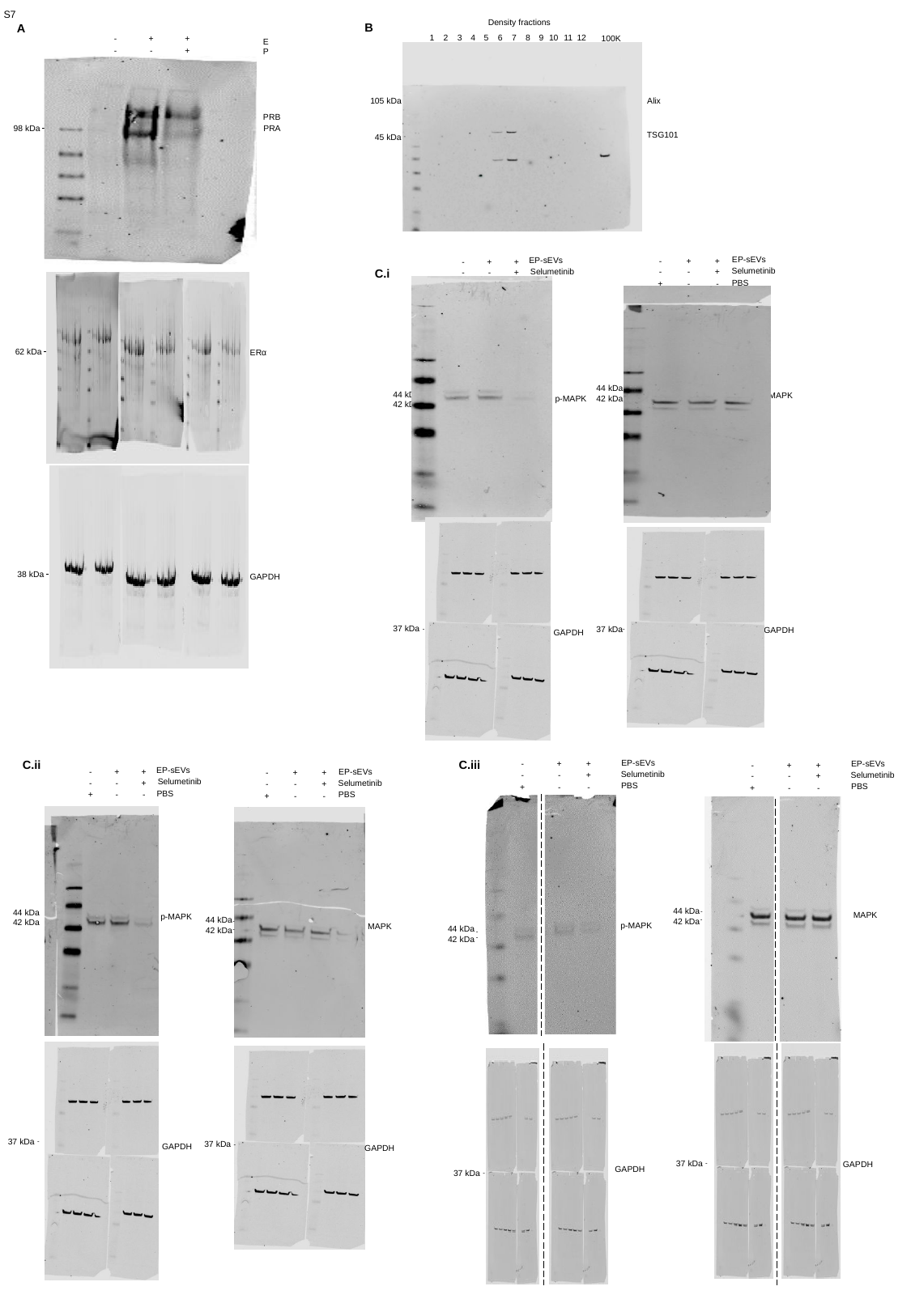

S7
Density fractions
B
A
1
2
3
4
5
6
7
8
9
10
11
12
100K
105 kDa
Alix
TSG101
45 kDa
-
+
+
-
-
+
E
P
 PRB
98 kDa
 PRA
EP-sEVs
-
+
+
Selumetinib
-
-
+
PBS
+
-
-
EP-sEVs
-
+
+
Selumetinib
-
-
+
PBS
+
-
-
44 kDa
42 kDa
-
MAPK
-
44 kDa
42 kDa
-
p-MAPK
-
37 kDa
-
GAPDH
37 kDa
GAPDH
-
C.i
62 kDa
ERα
38 kDa
GAPDH
C.ii
EP-sEVs
Selumetinib
PBS
EP-sEVs
Selumetinib
PBS
-
-
+
+
-
-
+
+
-
-
-
+
+
-
-
+
+
-
44 kDa
42 kDa
-
MAPK
-
p-MAPK
44 kDa
42 kDa
-
-
37 kDa
-
GAPDH
GAPDH
37 kDa
-
C.iii
EP-sEVs
-
+
+
Selumetinib
-
-
+
PBS
+
-
-
EP-sEVs
-
+
+
Selumetinib
-
-
+
PBS
+
-
-
44 kDa
42 kDa
p-MAPK
44 kDa
42 kDa
-
MAPK
-
37 kDa
-
GAPDH
37 kDa
-
GAPDH
